# Effect of Temperature, Oil Type, and Copolymer Concentration on the Long-Term Stability of Oil-in-Water Pickering Nanoemulsions Prepared Using Diblock Copolymer Nanoparticles

**DOI:** 10.1021/acs.langmuir.3c03423

**Published:** 2024-02-05

**Authors:** Saul J. Hunter, Priyanka Chohan, Spyridon Varlas, Steven P. Armes

**Affiliations:** † Department of Chemistry, University of Sheffield, Dainton Building, Brook Hill, Sheffield S3 7HF, South Yorkshire, U.K.; ‡ School of Chemistry, Joseph Banks Laboratories, University of Lincoln, Brayford Pool, Lincoln LN6 7TS, U.K.

## Abstract

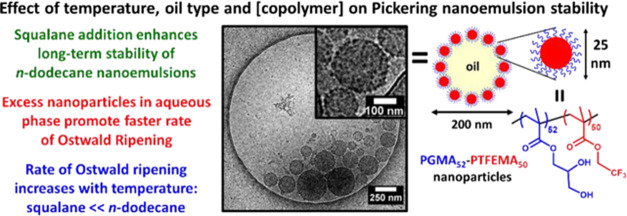

A poly(glycerol monomethacrylate) (PGMA) precursor was chain-extended with 2,2,2-trifluoroethyl methacrylate (TFEMA) via reversible addition–fragmentation chain transfer (RAFT) aqueous emulsion polymerization. Transmission electron microscopy (TEM) studies confirmed the formation of well-defined PGMA_52_–PTFEMA_50_ spherical nanoparticles, while dynamic light scattering (DLS) studies indicated a *z*-average diameter of 26 ± 6 nm. These sterically stabilized diblock copolymer nanoparticles were used as emulsifiers to prepare oil-in-water Pickering nanoemulsions: either *n*-dodecane or squalane was added to an aqueous dispersion of nanoparticles, followed by high-shear homogenization and high-pressure microfluidization. The Pickering nature of such nanoemulsion droplets was confirmed via cryo-transmission electron microscopy (cryo-TEM). The long-term stability of such Pickering nanoemulsions was evaluated by analytical centrifugation over a four-week period. The *n*-dodecane droplets grew in size significantly faster than squalane droplets: this is attributed to the higher aqueous solubility of the former oil, which promotes Ostwald ripening. The effect of adding various amounts of squalane to the *n*-dodecane droplet phase prior to emulsification was also explored. The addition of up to 40% (v/v) squalane led to more stable nanoemulsions, as judged by analytical centrifugation. The nanoparticle adsorption efficiency at the *n*-dodecane–water interface was assessed by gel permeation chromatography when using nanoparticle concentrations of 4.0, 7.0, or 10% w/w. Increasing the nanoparticle concentration not only produced smaller droplets but also reduced the adsorption efficiency, as confirmed by TEM studies. Furthermore, the effect of varying the nanoparticle concentration (2.5, 5.0, or 10% w/w) on the long-term stability of *n*-dodecane-in-water Pickering nanoemulsions was explored over a four-week period. Nanoemulsions prepared at higher nanoparticle concentrations were more unstable and exhibited a faster rate of Ostwald ripening. The nanoparticle adsorption efficiency was monitored for an aging nanoemulsion prepared at a copolymer concentration of 2.5% w/w. As the droplets ripened over time, the adsorption efficiency remained constant (∼97%). This suggests that nanoparticles desorbed from the shrinking smaller droplets and then readsorbed onto larger droplets over time. Finally, the effect of temperature on the stability of Pickering nanoemulsions was examined. Storing these Pickering nanoemulsions at elevated temperatures led to faster rates of Ostwald ripening, as expected.

## Introduction

Pickering emulsions are oil or water droplets that are stabilized by solid particles, such as silica, clays, or latexes.^1−3^ Such emulsions offer various advantages over conventional surfactant-stabilized emulsions, including greater long-term stability, more reproducible formulations, reduced foaming problems, and lower toxicity.^3^ Unlike surfactant molecules, particles are normally considered to be irreversibly adsorbed at the oil–water interface, which reduces the interfacial area and hence lowers the free energy. The surface wettability of the particles dictates whether oil-in-water (O/W) or water-in-oil (W/O) Pickering emulsions are formed.^3−5^

Pickering nanoemulsions comprise either oil or water droplets of 50–500 nm diameter that are stabilized using nanoparticles.^6−15^ They are readily prepared via high-pressure microfluidization of Pickering macroemulsions provided that sufficient excess nanoparticles are present to stabilize the additional surface area that is generated when producing much finer droplets.^6,16^ The final droplet diameter typically depends on both the applied pressure and the number of passes through a commercial microfluidizer.^16^ Under optimized conditions, droplet diameters of less than 200 nm can be achieved.^6,16−19^ Given their much higher surface area per unit mass, nanoemulsions offer more active formulations than conventional emulsions, which may be useful for applications in cosmetics,^20^ drug delivery,^21^ agrochemicals,^22,23^ and food technology.^24,25^

Pickering nanoemulsions are relatively insensitive to gravitational creaming (or sedimentation) owing to their relatively small droplet size. However, their relatively high surface area makes them prone to droplet growth via Ostwald ripening.^15,26−42^ For O/W nanoemulsions, This involves the diffusion of oil molecules from smaller droplets through the aqueous continuous phase to larger droplets over time. Although there are many literature reports on the formation of copolymer- or surfactant-stabilized nanoemulsions, there are surprisingly few studies focused on Pickering nanoemulsions.^6−9,12−18,43−46^ This is no doubt because the Pickering emulsifier should be typically 5–10 times smaller than the mean droplet diameter.^16^ Thus, droplets of (say) 200 nm diameter require nanoparticles of 20–40 nm diameter. With the exception of silica sols, such nanoparticles are not widely available as concentrated dispersions. Moreover, they must also be capable of surviving high-shear homogenization and high-pressure microfluidization.^13,18^

Polymerization-induced self-assembly (PISA) offers an efficient synthetic route to a wide range of diblock copolymer nanoparticles of tunable surface wettability.^47−54^ Typically, aqueous PISA formulations involve growing a water-insoluble polymer chain from one end of a water-soluble polymer precursor.^19,48,55−62^ The resulting amphiphilic diblock copolymer chains undergo *in situ* micellar nucleation to produce a concentrated colloidal dispersion of sterically stabilized nanoparticles. The most common copolymer morphology is spheres and the mean diameter can be as low as 10–20 nm diameter.^56,60,63−65^ Thus aqueous PISA enables the convenient synthesis of hydrophilic spherical nanoparticles that are suitable for the preparation of Pickering nanoemulsions.^13,16−18,27,43^

For example, Thompson and co-workers examined the relative stability of a series of *n*-alkane-in-water Pickering nanoemulsions prepared using sterically stabilized nanoparticles comprising a hydrophilic poly(glycerol monomethacrylate) (PGMA) block and a hydrophobic poly(2,2,2-trifluoroethyl methacrylate) (PTFEMA) block.^16,27^ Analytical centrifugation studies confirmed that nanoemulsions prepared using oils with higher aqueous solubility (e.g., *n*-octane or *n*-decane) were more susceptible toward Ostwald ripening during storage for up to 7 days at 20 °C. Conversely, nanoemulsions prepared using either *n*-dodecane or *n*-tetradecane exhibited minimal droplet growth under the same conditions.

Recently, we explored the effect of varying the nanoparticle concentration on the long-term stability of *n*-dodecane-in-water Pickering nanoemulsions, which were prepared using either 5 or 10% w/w poly(*N,N*′-dimethylacrylamide)-poly(diacetone acrylamide) (PDMAC_77_–PDAAM_40_) nanoparticles.^18^ Analytical centrifugation studies indicated a gradual evolution from unimodal to bimodal droplet size distributions on storage for 8 weeks at 20 °C, with a more rapid increase in droplet size being observed for nanoemulsions prepared at higher nanoparticle concentrations. This was attributed to more efficient mass transport of oil molecules through the aqueous phase mediated by excess (non-adsorbed) nanoparticles.

Herein we explore the effect of varying the temperature, nanoparticle concentration, and oil type on the rate of Ostwald ripening of a series of oil-in-water Pickering nanoemulsions prepared using PGMA_52_–PTFEMA_50_ nanoparticles to stabilize either *n*-dodecane or squalane droplets.

## Experimental Section

### Materials

All reagents were used as received, unless otherwise stated. Glycerol monomethacrylate (GMA; 99.8% purity) was obtained from GEO Specialty Chemicals (Hythe, U.K.). 2,2,2-Trifluoroethyl methacrylate (TFEMA), 4,4′-azobis(4-cyanopentanoic acid) (ACVA), *n*-dodecane, squalane, and ethanol were all purchased from Sigma-Aldrich (U.K.). Each monomer was passed through a basic alumina column to remove its inhibitor prior to use. 2-Cyano-2-propyl dithiobenzoate (CPDB) was purchased from Strem Chemicals Ltd. (Cambridge, U.K.). *d*_6_-Acetone and *d*_4_-methanol (CD_3_OD) were purchased from Goss Scientific Instruments Ltd. (Cheshire, U.K.). *N,N-*Dimethylformamide (DMF) was purchased from Fisher Scientific (U.K.). Deionized water (pH 6) was used for all studies involving aqueous solutions.

### Methods

#### Synthesis of a PGMA_52_ Precursor via RAFT Solution Polymerization in Ethanol

GMA (30.1 g, 0.186 mol), CPDB (0.589 g, 2.66 mmol; target DP = 70), ACVA (0.149 g, 0.532 mmol; CPDB/ACVA molar ratio = 5.0), and anhydrous ethanol (45.9 g) were weighed into a 100 mL round-bottom flask. The resulting solution was deoxygenated by purging with a stream of nitrogen gas for 30 min at 20 °C before the flask was immersed in an oil bath at 70 °C for 165 min with continuous stirring. The polymerization was quenched by removal of the flask from the oil bath and exposure of the reaction mixture to air while cooling the flask to 20 °C. A GMA conversion of 72% was determined by ^1^H NMR spectroscopy by comparing the integrated monomer vinyl signals at 5.7 and 6.2 ppm with the five pendent PGMA proton signals at 3.7–4.3 ppm. The crude polymer was purified by precipitation into a ten-fold excess of DCM (three times) and then freeze-dried from water. A mean DP of 52 was determined via end-group analysis using ^1^H NMR spectroscopy (*d*_4_-methanol) by comparing the integrated peaks of the aromatic protons assigned to the dithiobenzoate chain ends at 7.4–7.9 ppm to the five pendent GMA protons at 3.7–4.3 ppm. DMF GPC analysis (using a UV detector set at 298 nm) indicated an *M*_n_ of 13 100 g mol^–1^ and an *M*_w_/*M*_n_ of 1.21.

#### Synthesis of PGMA_52_–PTFEMA_50_ Diblock Copolymer Nanoparticles via RAFT Aqueous Emulsion Polymerization of TFEMA

A PGMA_52_ precursor (2.50 g, 0.304 mmol), ACVA (0.0167 g, 0.0608 mmol; PGMA_52_/ACVA molar ratio = 5.0), and deionized water (45.6 g) were added to a 100 mL round-bottom flask, and then degassed with a stream of nitrogen gas for 30 min at 20 °C. TFEMA (2.16 mL, 0.0152 mol; target DP = 50 at 10% w/w solids) was degassed separately using an ice bath to minimize evaporation and then injected into the round-bottom flask prior to its immersion in an oil bath at 70 °C for 6 h. The TFEMA polymerization was quenched by exposing the reaction mixture to air while cooling the flask to ambient temperature. ^19^F NMR spectroscopy analysis in *d*_6_-acetone indicated more than 99% TFEMA conversion. DMF GPC analysis (using a UV detector set at 298 nm) indicated an *M*_n_ of 22 000 g mol^–1^ and an *M*_w_/*M*_n_ of 1.19.

#### Preparation of PGMA_52_–PTFEMA_50_-Stabilized Pickering Macroemulsions using High-Shear Homogenization

An aqueous dispersion of PGMA_52_–PTFEMA_50_ nanoparticles (2.40 mL, 7.0% w/w) was added to a 14 mL glass vial and homogenized with *n*-dodecane (0.60 mL) at 13 500 rpm for 2 min at 20 °C using an IKA Ultra-Turrax T-18 homogenizer equipped with a 10 mm dispersing tool. The same protocol was used to prepare squalane-in-water Pickering macroemulsions. A series of Pickering macroemulsions were also prepared by systematically varying the nanoparticle concentration from 2.5 to 10% w/w.

#### Preparation of PGMA_52_–PTFEMA_50_-Stabilized Pickering Nanoemulsions using High-Pressure Microfluidization

A Pickering macroemulsion (3.0 mL) was further processed with the aid of an LV1 microfluidizer (Microfluidics). The applied pressure was 20 000 psi, and each macroemulsion was passed through the LV1 ten times to afford well-defined Pickering nanoemulsions.

#### NMR Spectroscopy

All ^1^H and ^19^F NMR spectra were recorded in either *d*_4_-methanol or *d*_6_-acetone using a Bruker Avance-400 spectrometer operating at 400 MHz.

#### Gel Permeation Chromatography (GPC)

An Agilent 1260 Infinity GPC system equipped with a differential refractive index detector and a UV detector was used to determine the number-average molecular weight (*M*_n_), weight-average molecular weight (*M*_w_), and dispersity (*M*_w_/*M*_n_) for each (co)polymer. Two Agilent PL-gel 5 μm mixed-C columns and a guard column were connected in series to this GPC system. Unless otherwise stated, high-performance liquid chromatography (HPLC) grade DMF containing 10 mM LiBr was used as the eluent. GPC analysis was performed at 60 °C using a constant flow rate of 1.0 mL min^–1^. A series of near-monodisperse poly(methyl methacrylate) calibration standards with *M*_p_ values ranging from 800 g mol^–1^ to 2 200 000 g mol^–1^ were used to calculate molecular weights and dispersities. All (co)polymer samples were diluted to 1.0% w/w using the GPC eluent and chromatograms were analyzed using Agilent GPC/SEC software.

#### Dynamic Light Scattering (DLS)

Intensity-average size distributions and z-average hydrodynamic diameters, *D_z_*, were obtained at a scattering angle of 173° using a Malvern Zetasizer Nano ZS instrument. Dilute (0.1% w/w) dispersions of PGMA_52_–PTFEMA_50_ nanoparticles and various Pickering nanoemulsions were analyzed by using disposable plastic cuvettes at 20 °C. In each case, the results were averaged over three consecutive runs.

#### Transmission Electron Microscopy (TEM)

Copper/palladium TEM grids (Agar Scientific, U.K.) were surface-coated with a thin film of amorphous carbon. If required, grids were subjected to a plasma glow discharge for 30 s to produce a hydrophilic surface. One droplet of an aqueous dispersion of nanoparticles (0.2% w/w, 10 μL) or a nanoemulsion (0.5% v/v, 10 μL) was placed on a grid for 1 min, after which any remaining solution was removed by blotting with filter paper. Subsequently, an aqueous droplet of uranyl formate (0.75% w/w, 10 μL) was placed on the sample-loaded grid for 20 s, and the excess stain was removed by blotting. Each grid was carefully dried using a vacuum hose. Images were recorded using an FEI Tecnai Spirit microscope operating at 80 kV and equipped with a Gatan 1kMS600CW CCD camera.

#### Cryogenic Transmission Electron Microscopy (cryo-TEM)

Imaging was performed using an FEI Tecnai Arctica microscope operating at an acceleration voltage of 200 kV. Cryo-TEM samples were prepared by depositing 5 μL of a 0.5% w/w dodecane-in-water or squalane-in-water Pickering nanoemulsion onto a plasma-treated Quantifoil holey carbon-coated copper grid, followed by blotting for approximately 4 s and then plunging into a pool of liquid ethane to vitrify the sample using a Leica EM GP automatic plunge freezer (25 °C, 99% humidity). Transfer of the vitrified grids into a precooled cryo-TEM holder was performed at −196 °C prior to microscopic analysis.

#### Analytical Centrifugation (LUMiSizer)

Droplet size distributions were analyzed using a LUMiSizer analytical photocentrifuge (LUM GmbH, Berlin, Germany) at 20–60 °C. Measurements were conducted on 1.0% v/v Pickering nanoemulsions using 2 mm path length polyamide cells at 500, 1000, 2000, and 4000 rpm for 200 profiles each (with 10 s between each profile). The LUMiSizer instrument uses space- and time-resolved extinction profiles (STEP) technology to measure the intensity of transmitted near-infrared light as a function of time and position over the entire cell length. The gradual progression of transmission profiles contained information on the rate of creaming, which allowed assessment of the droplet size distribution. The droplet density is an essential input parameter for analytical centrifugation measurements. For the nanoemulsion aging studies, this parameter was taken to be either 0.75 g cm^–3^ (for *n*-dodecane droplets) or 0.81 g cm^–3^ (for squalane droplets). Such densities ignore the contribution from the relatively dense PGMA_52_–PTFEMA_50_ nanoparticles adsorbed at the surface of each oil droplet. However, this approximation is acceptable for the present study, given that only relative changes in the droplet size distribution are assessed over time.

#### Small-Angle X-ray Scattering (SAXS)

SAXS experiments were conducted on 1.0% w/w aqueous dispersions of PGMA_52_–PTFEMA_50_ nanoparticles and the corresponding 1.0% v/v squalane-in-water Pickering nanoemulsion at the ESRF (station ID02, Grenoble, France) using monochromatic X-ray radiation (λ = 0.0995 nm; *q* range = 0.002–0.15 Å^–1^, where *q* is the length of the scattering vector and θ is one-half of the scattering angle, such that *q* = 4π sin θ/λ) and an Eiger24M two-dimensional detector (Dectris, Switzerland). A flow-through glass capillary (2 mm diameter) was connected to a syringe and a waste container via plastic tubing and mounted horizontally on the beamline stage; this setup was used as a sample holder. Scattering data were reduced using standard routines provided by the beamline and were further analyzed using Irena SAS macros for Igor Pro.^66^

## Results and Discussion

### Synthesis and Characterization of PGMA_52_–PTFEMA_50_ Diblock Copolymer Nanoparticles

A PGMA precursor with a target DP of 70 was prepared via RAFT solution polymerization in ethanol using a CPDB RAFT agent and an ACVA initiator. To ensure retention of the end-group fidelity, this polymerization was quenched after 165 min, which produced a GMA conversion of 72%. After purification of the crude polymer, its mean DP was determined to be 52 by end-group analysis using ^1^H NMR spectroscopy (see Figure S1). DMF GPC studies of this precursor indicated an *M*_n_ of 13 100 g mol^–1^ and an *M*_w_/*M*_n_ of 1.21, as shown in Figure S3.

**Figure 1:**
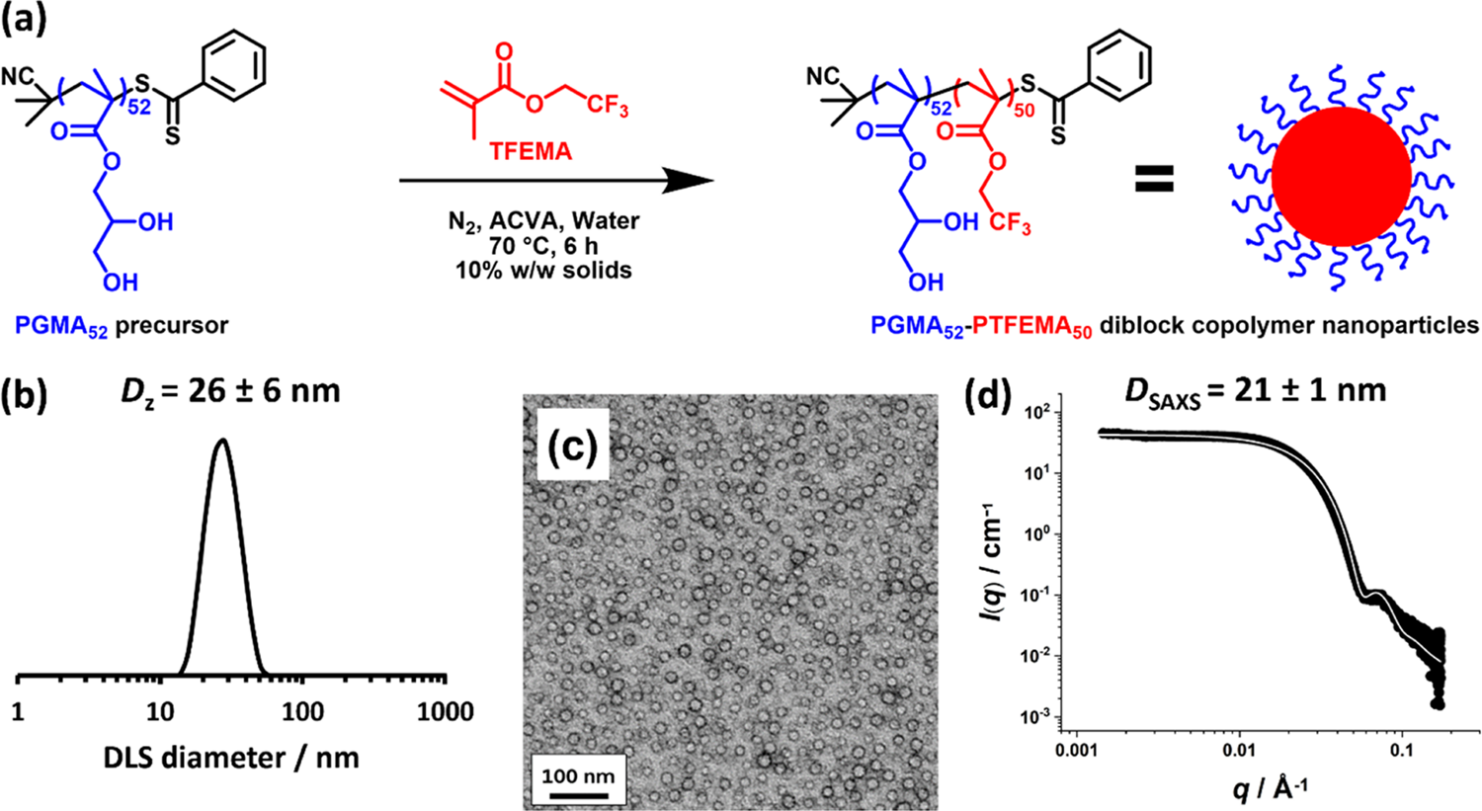
(a) Synthesis of PGMA_52_–PTFEMA_50_ diblock copolymer spheres via the RAFT aqueous emulsion polymerization of TFEMA at 70 °C. (b) DLS intensity-average particle size distribution obtained for a 0.1% w/w aqueous dispersion of PGMA_52_–PTFEMA_50_ diblock copolymer nanoparticles. (c) TEM image recorded after drying a 0.2% w/w aqueous dispersion of PGMA_52_–PTFEMA_50_ nanoparticles at 20 °C. (d) SAXS pattern recorded for a 1.0% w/w aqueous dispersion of PGMA_52_–PTFEMA_50_ nanoparticles at 20 °C. The data fit (solid white line) was obtained using a spherical micelle model.^67,68^

PGMA_52_–PTFEMA_50_ diblock copolymer nanoparticles were prepared by chain-extending the water-soluble PGMA_52_ precursor via RAFT aqueous emulsion polymerization of TFEMA using a protocol reported by Akpinar and co-workers.^45^ The synthesis of such nanoparticles is outlined in Figure 1a. ^19^F NMR spectroscopy analysis of the resulting diblock copolymer indicated more than 99% TFEMA conversion (the integrated residual TFEMA monomer signal at −74.34 ppm was compared to the signal assigned to the PTFEMA block at −73.95 ppm; see Figure S2). DMF GPC studies of this diblock copolymer indicated an *M*_n_ of 22 000 g mol^–1^ and a relatively narrow molecular weight distribution (*M*_w_/*M*_n_ = 1.19); see Figure S3. The unimodal GPC trace indicates efficient chain extension and hence minimal contamination from the unreacted PGMA_52_ precursor.

DLS characterization of the nanoparticles indicated a z-average hydrodynamic diameter of 26 ± 6 nm and a relatively low DLS polydispersity of 0.05 (Figure 1b). TEM analysis confirmed a well-defined spherical morphology (Figure 1c). Since this technique is conducted under ultrahigh vacuum, only the PTFEMA cores can be observed. Digital image analysis indicated an approximate number-average diameter of 18 ± 2 nm for a population of 100 nanoparticles. Small-angle X-ray scattering (SAXS) studies reported an overall volume-average diameter of 21 ± 1 nm (Figure 1d).

### Effect of Oil Solubility on the Stability of Pickering Nanoemulsions

**Figure 2:**
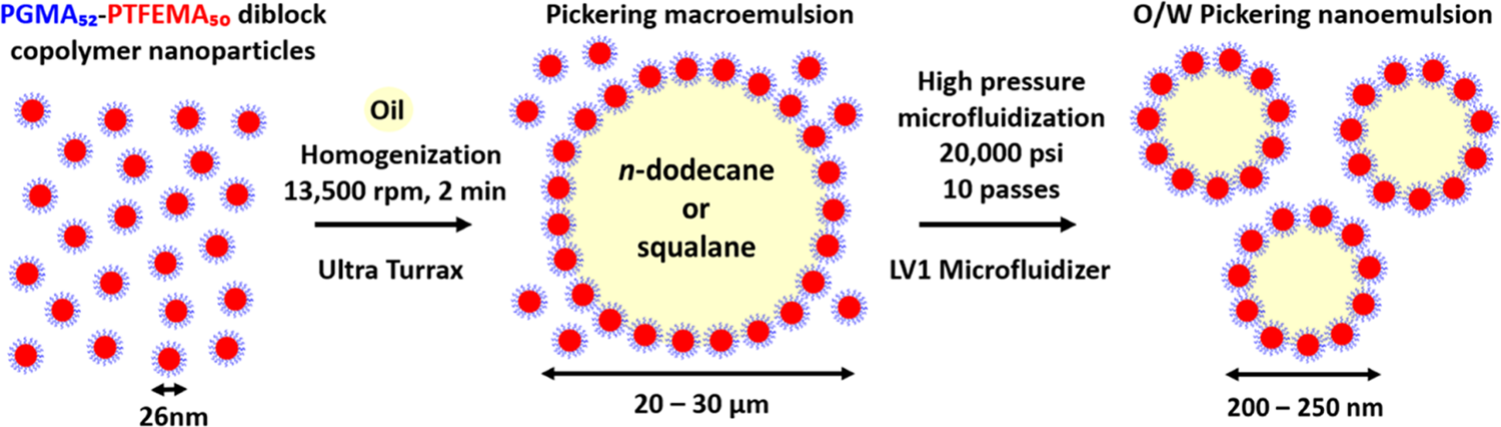
Schematic representation for the formation of O/W Pickering nanoemulsions using PGMA_52_–PTFEMA_50_ nanoparticles as a Pickering emulsifier. First, high-shear homogenization of 2.5–10% w/w aqueous dispersions of such nanoparticles with 20% v/v *n*-dodecane (or squalane) resulted in the formation of a Pickering macroemulsion with a mean droplet diameter of 20–30 μm. Then this precursor macroemulsion was passed ten times through a high-pressure microfluidizer at 20 000 psi to produce the desired O/W Pickering nanoemulsion with a mean droplet diameter of approximately 200−250 nm.

Pickering macroemulsions were prepared by homogenizing 2.5–10% w/w aqueous dispersions of PGMA_52_–PTFEMA_50_ nanoparticles with 20% v/v *n*-dodecane (or squalane) for 2 min at 13 500 rpm. This produced relatively coarse oil droplets with a mean volume-average diameter of 20–30 μm. These macroemulsions were then passed ten times through a high-pressure microfluidizer at 20 000 psi, resulting in the formation of Pickering nanoemulsions of approximately 200 nm diameter. This processing step is illustrated in Figure 2. The oil volume was fixed at 20% because higher volumes might cause either phase inversion^76^ or produce unstable emulsion droplets.^77^ Thompson et al. reported that an applied pressure of 20 000 psi was optimal for the preparation of stable o/w Pickering nanoemulsions when using similar PGMA_48_–PTFEMA_50_ nanoparticles.^70^ Lower pressures produced larger, more polydisperse droplets, whereas higher pressures caused *in situ* nanoparticle disassembly, which led to droplets becoming stabilized by individual amphiphilic copolymer chains (rather than intact nanoparticles). Thompson et al. also found that at least eight passes through the microfluidizer were required to achieve a unimodal size distribution, with ten passes producing the smallest droplets. Hence, this previously optimized protocol was used to produce all of the O/W Pickering nanoemulsions reported in the present study.

In 2010, Persson et al. used commercially available silica nanoparticles to prepare a series of O/W Pickering nanoemulsions with various oils.^6^ However, *n*-alkanes (including *n*-dodecane) invariably led to highly unstable droplets; only squalene (a naturally occurring, highly water-insoluble oil) produced relatively stable droplets. Thompson and co-workers subsequently investigated the long-term stability of a series of O/W Pickering nanoemulsions prepared using PGMA_48_–PTFEMA_50_ nanoparticles to stabilize *n*-octane, *n*-decane, *n*-dodecane, or *n*-tetradecane droplets.^71^ Increasing the *n*-alkyl chain length of the oil significantly lowered its solubility in the aqueous phase and, as a result, reduced the rate of Ostwald ripening.^71^

**Figure 3:**
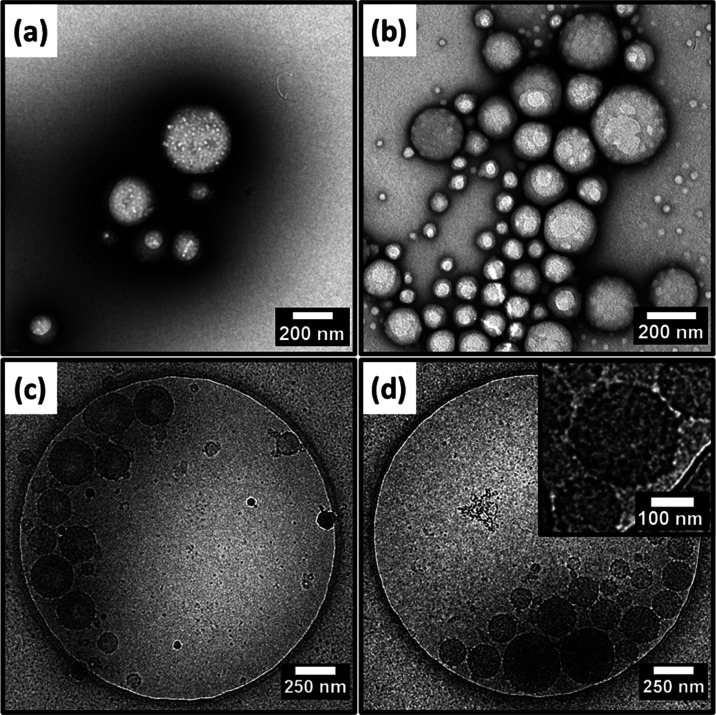
Representative (a, b) conventional TEM and (c, d) cryo-TEM images obtained for freshly prepared (a, c) *n*-dodecane-in-water or (b, d) squalane-in-water Pickering nanoemulsions prepared via high-pressure microfluidization using 7.0% w/w PGMA_52_–PTFEMA_50_ nanoparticles and 20% v/v oil. Nanoemulsion preparation conditions: applied pressure = 20 000 psi for 10 passes using an LV1 microfluidizer.

Accordingly, two Pickering nanoemulsions were prepared by using a 7.0% w/w aqueous dispersion of PGMA_52_–PTFEMA_50_ nanoparticles and 20% v/v *n*-dodecane (or squalane) for TEM studies. Representative TEM images obtained for freshly prepared nanoemulsions are shown in Figure 3a,b. Both the oil droplet phase and the aqueous continuous phase evaporate completely under ultrahigh vacuum conditions, leaving only the nanoparticles that were adsorbed at the surface of the oil droplets. These spherical nanoparticle superstructures are comparable in size to the mean DLS diameter of the original oil droplets. Furthermore, close inspection reveals the presence of the individual nanoparticles, which confirms the genuine Pickering nature of such nanoemulsions. Finally, there are relatively few nanoparticles remaining in the background, indicating a relatively high adsorption efficiency.

Cryo-TEM images obtained for the same two Pickering nanoemulsions are shown in Figure 3c,d. For this imaging technique, both the oil droplets and the aqueous continuous phase are subjected to rapid freezing rather than evaporation. Again, the frozen droplets have mean diameters that are comparable to those reported by DLS and there is also some evidence for the presence of adsorbed nanoparticles at the droplet surface.

**Figure 4:**
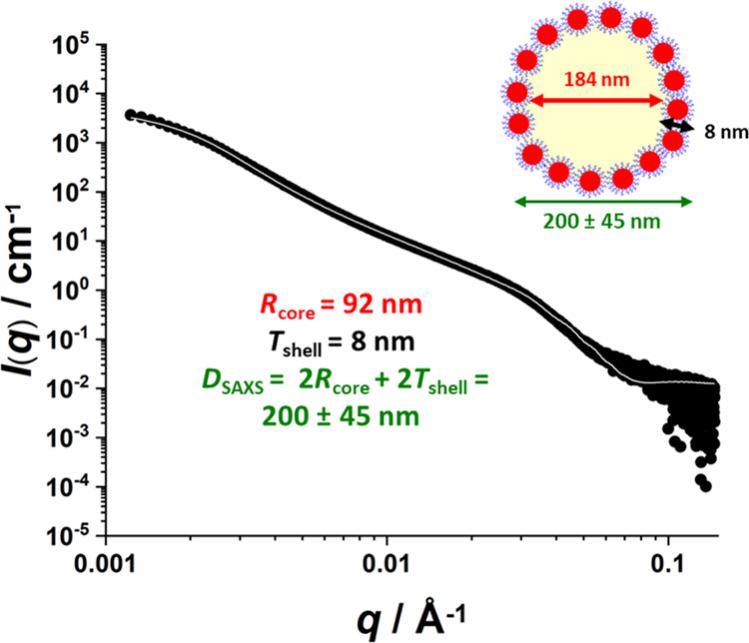
SAXS pattern (black circles) and corresponding data fit (solid white line) obtained for a 1.0% v/v Pickering nanoemulsion prepared using 7.0% w/w PGMA_52_–PTFEMA_50_ nanoparticles with squalane as the oil phase. The two-population core–shell structural model^43^ used for SAXS analysis of this Pickering nanoemulsion comprises squalane droplet cores coated with an adsorbed layer of PGMA_52_–PTFEMA_50_ spherical nanoparticles. Nanoemulsion preparation conditions: applied pressure = 20 000 psi for 10 passes using an LV1 microfluidizer.

To calculate the adsorption efficiency of the nanoparticles on the oil droplets, a calibration plot of integrated UV GPC signal intensity (recorded at λ = 298 nm) against nanoparticle concentration was constructed (see Figure S4).^43^ This involved analyzing the UV chromatograms recorded after serial dilution of an aqueous dispersion of PGMA_52_–PTFEMA_50_ nanoparticles using DMF. This is a good solvent for both PGMA and PTFEMA and hence results in nanoparticle dissolution to form soluble diblock copolymer chains. This linear calibration plot was used to determine the concentration of non-adsorbed nanoparticles remaining in the aqueous supernatant after centrifugation of the nanoemulsion at 13 000 rpm for 15 min. This causes creaming (rather than sedimentation) of the oil droplets. Subtracting the concentration of non-adsorbed nanoparticles from the initial nanoparticle concentration enables calculation of the adsorption efficiency, *A*_eff_, which was determined to be 97% for a freshly prepared *n*-dodecane-in-water nanoemulsion. Next, the mean packing efficiency, *P*, for the adsorbed layer of nanoparticles surrounding each oil droplet was calculated using a core–shell model reported by Balmer et al.^69^ Assuming a contact angle of 0° for nanoparticle adsorption at the oil–water interface, *P* was determined to be 50% for a Pickering nanoemulsion prepared using *n*-dodecane at a PGMA_52_–PTFEMA_50_ concentration of 7% w/w. A 1.0% v/v squalane-in-water nanoemulsion was also characterized using SAXS. The resulting SAXS pattern (see Figure 4) was analyzed using the same two-population model (see the Supporting Information for further details) previously used to characterize *n*-dodecane-in-water and water-in-*n*-dodecane Pickering nanoemulsions.^13,43^

One population is represented by core–shell spheres, where the cores comprise oil droplets and the shell is formed by the adsorbed layer of nanoparticles. The second population corresponds to the particulate nature of the shell and is described by spherical micelles with a hard-sphere structure factor to account for interparticle interactions at the oil–water interface. This two-population model produced a satisfactory fit to the scattering pattern (see Figure 4) and an overall mean droplet radius of 92 ± 22 nm, which is consistent with the corresponding DLS data (*D*_z_ = 196 ± 63 nm). The mean apparent shell thickness for the adsorbed layer of nanoparticles was calculated to be approximately 8 nm, which is significantly lower than the volume-average diameter of an individual nanoparticle (∼21 ± 1 nm; Figure 1d). However, this is consistent with the relatively low surface coverage of the oil droplets by the nanoparticles, which exhibit a packing efficiency of around 40%.

**Figure 5:**
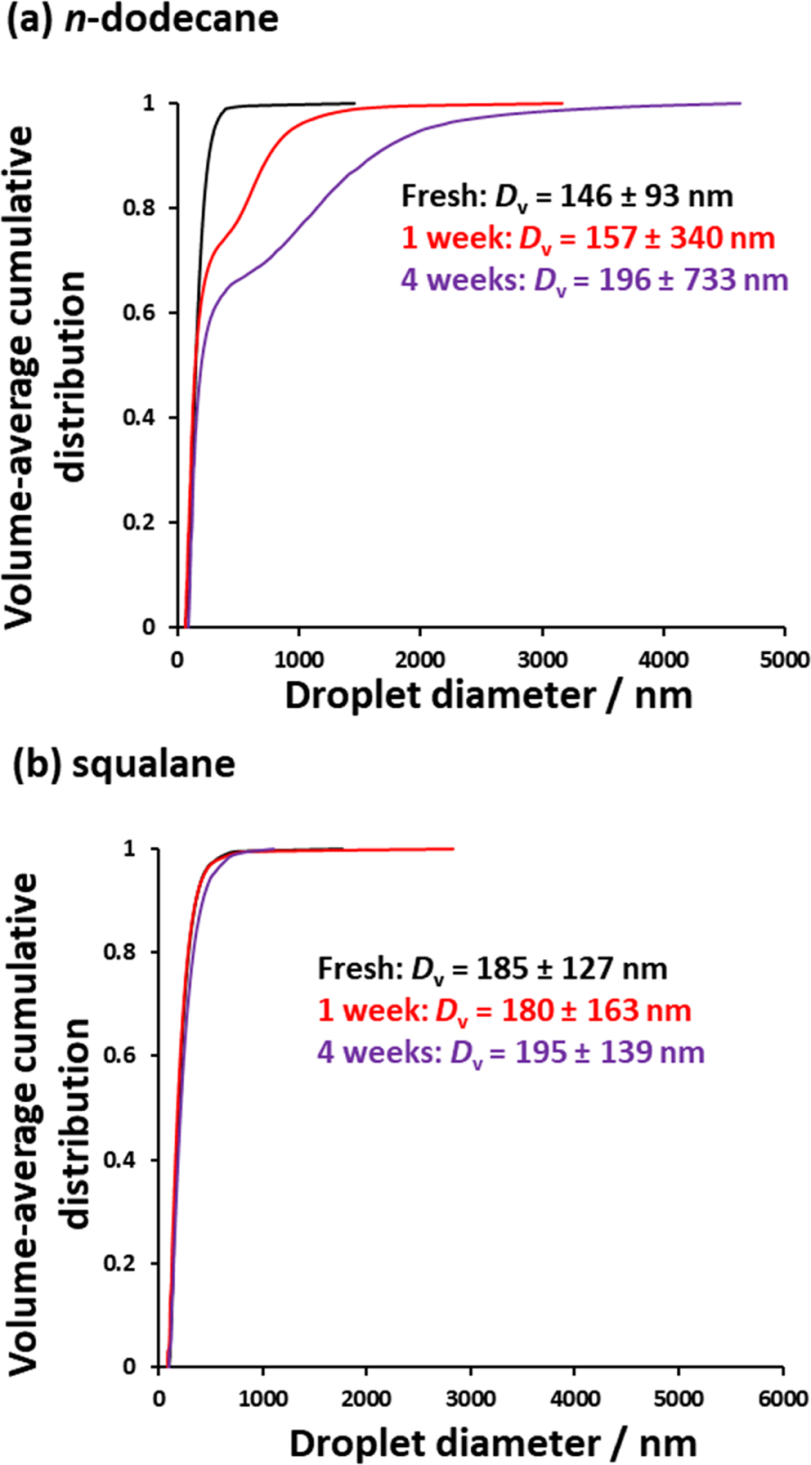
Volume-average cumulative distributions determined by analytical centrifugation after aging for up to 4 weeks at 20 °C: (a) an *n*-dodecane-in-water nanoemulsion and (b) a squalane-in-water nanoemulsion at 20% v/v. The harmonic mean ± standard deviation is reported for each time period. Nanoemulsion preparation conditions: applied pressure = 20 000 psi for 10 passes using an LV1 microfluidizer.

The droplet size distribution of each nanoemulsion was monitored over the course of four weeks via analytical centrifugation (LUMiSizer instrument). Analytical centrifugation was preferred to DLS for the study of droplet growth because it is a high-resolution technique, with oil droplets being fractionated according to their size prior to detection.^71^ The data obtained from such analytical centrifugation studies are depicted in Figure 5. For such experiments, the standard deviation of the droplet size distribution is perhaps a more sensitive indicator of the relative droplet stability than the mean diameter.

Inspecting Figure 5, it is evident that the *n*-dodecane droplet size distribution becomes significantly broader after aging for 4 weeks at 20 *°*C. In contrast, only minimal change is observed for the analogous squalane droplets over the same time scale. This can be explained by Lifshitz–Slyozov–Wagner (LSW) theory for Ostwald ripening.^34,37,70,71^ This assumes that (i) the oil phase comprises solely spherical droplets, (ii) the distance between neighboring droplets is substantially larger than the mean droplet diameter, and (iii) droplet growth is solely governed by the diffusion of oil (or water) molecules through the aqueous (or oil) phase.^79^ The rate of Ostwald ripening, ω, can be calculated using eq 1, where *C*(∞) is the solubility of the oil phase within the aqueous phase, γ is the interfacial tension, *V*_m_ is the molar volume of the oil, *D* is the diffusion coefficient for the oil droplets within the aqueous phase, and ρ is the density of the oil phase.
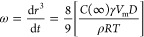
1

According to eq 1, the rate of Ostwald ripening is proportional to the solubility of the droplet phase within the continuous phase. Thus, using a highly water-insoluble oil such as squalane (aqueous solubility = 0.012 μg dm^–3^ at 25 °C^6^) should suppress the rate of Ostwald ripening much more significantly than when using *n*-dodecane (aqueous solubility = 3.4 μg dm^–3^ at 20 °C^27^).

**Figure 6:**
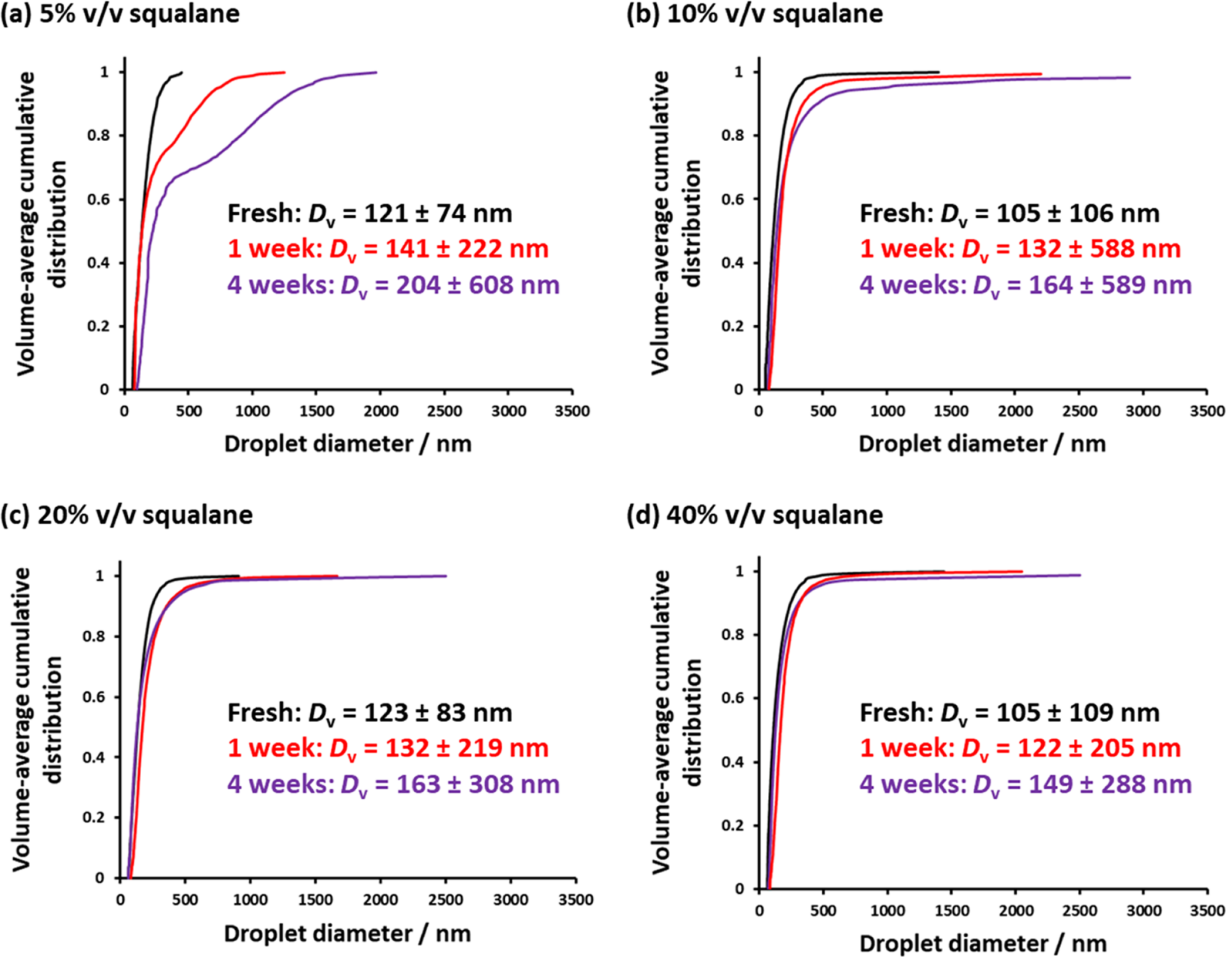
Volume-average cumulative distributions determined by analytical centrifugation after aging for up to 4 weeks at 20 °C: an *n*-dodecane-in-water nanoemulsion at 20% v/v containing (a) 5%, (b) 10%, (c) 20%, and (d) 40% squalane by volume. The harmonic mean ± standard deviation is reported for each time period. Nanoemulsion preparation conditions: applied pressure = 20 000 psi for 10 passes using an LV1 microfluidizer.

In principle, Ostwald ripening can be suppressed by adding a suitable species to the droplet phase that is highly insoluble in the continuous phase.^40,72^ For example, it is well-documented that the addition of a relatively long hydrocarbon (or wax) to oil droplets enhances the stability of O/W nanoemulsions toward Ostwald ripening.^29,34,40,73^ Similarly, the addition of salt to the aqueous phase is known to inhibit interdroplet mass transfer in the case of W/O emulsions.^13,74^ In the present study, varying proportions of squalane (5 to 40% by volume) were added to *n*-dodecane prior to high-shear homogenization. Using analytical centrifugation to monitor the evolution of droplet size distributions over time, we can examine how the presence of squalane affects the rate of Ostwald ripening of *n*-dodecane-in-water nanoemulsions (see Figure 6). Clearly, the addition of increasing amounts of squalane progressively retards the rate of Ostwald ripening. However, this enhanced stability never matches that achieved for Pickering nanoemulsions comprising squalane alone (see Figure 5).

### Effect of Copolymer Concentration on the Long-Term Stability of Pickering Nanoemulsions

Recently, Hunter and Armes reported that preparing Pickering nanoemulsions using higher copolymer concentrations led to a faster rate of Ostwald ripening, which was attributed to the excess nanoparticles facilitating oil transport through the aqueous phase.^18^

To extend this prior study, we decided to examine the effect of copolymer concentration on the adsorption efficiency, *A*_eff_, the number of nanoparticles per droplet, *N*, and the packing efficiency, *P*, was determined. Furthermore, the change in nanoparticle adsorption efficiency over time was examined by using a previously reported UV GPC protocol to monitor the nanoparticle concentration remaining in the aqueous continuous phase.^18^

**Figure 7:**
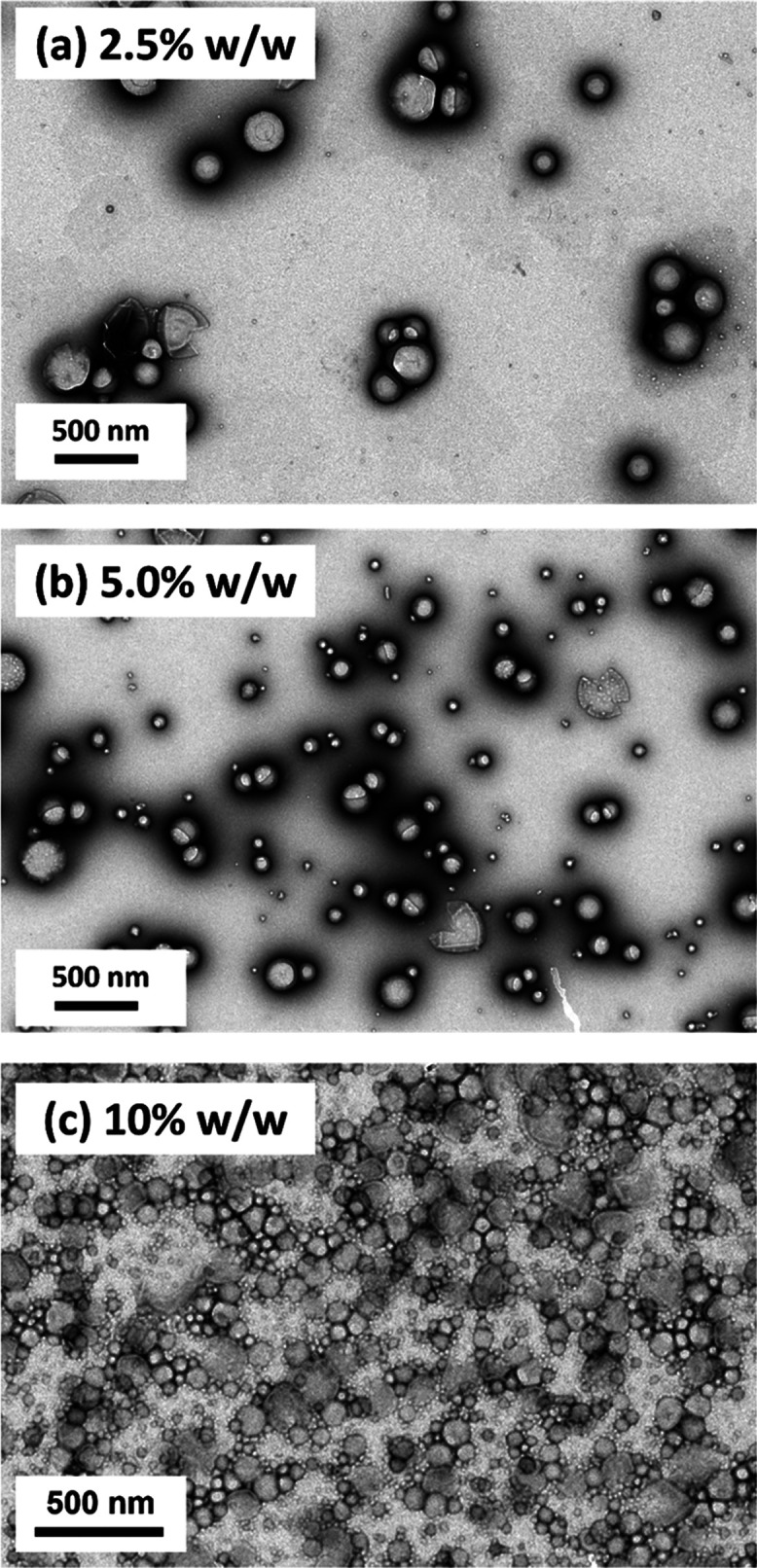
Representative TEM images recorded after drying freshly prepared n-dodecane-in-water Pickering nanoemulsions prepared using (a) 2.5% w/w, (b) 5.0% w/w, or (c) 10% w/w PGMA_52_–PTFEMA_50_ nanoparticles. Nanoemulsion preparation conditions: applied pressure = 20 000 psi for 10 passes using an LV1 microfluidizer.

First, the effect of varying the nanoparticle concentration on the adsorption efficiency was examined for freshly prepared and aged nanoemulsions. Accordingly, three *n*-dodecane-in-water Pickering nanoemulsions were prepared using 2.5, 5.0, or 10% w/w PGMA_52_–PTFEMA_50_ nanoparticles. TEM images were recorded after drying each of the three freshly prepared nanoemulsions to observe the original nanoparticle superstructure and assess the presence of any excess non-adsorbed nanoparticles (see Figure 7). Clearly, increasing the nanoparticle concentration leads to finer droplets but at the expense of a higher proportion of non-adsorbed nanoparticles. However, such TEM studies might be prone to drying artifacts arising during sample grid preparation. Thus nanoparticle adsorption efficiencies were determined for the freshly prepared nanoemulsions using UV GPC.^43^ Such experiments revealed that increasing the nanoparticle concentration reduced the adsorption efficiency; see Table 1. At lower nanoparticle concentrations, there are fewer nanoparticles available to stabilize the relatively large oil droplets, leading to an adsorption efficiency of almost 100%. At higher nanoparticle concentrations, smaller droplets are formed and the total surface area of the oil phase is higher. However, at some point the nanoparticles are present in excess. Thus some of the nanoparticles are no longer able to adsorb at the oil–water interface, which inevitably leads to lower adsorption efficiencies. As expected, there are fewer nanoparticles adsorbed per droplet, *N*, as the mean droplet diameter is reduced.

**Table 1:** Summary of the Characterization Data Obtained for the Three *n*-Dodecane-in-Water Pickering Nanoemulsions Prepared Using 2.5, 5.0, or 10% w/w PGMA_52_–PTFEMA_50_ Nanoparticles^a^

nanoparticle concentration (% w/w)	*D*_z_ (nm)	*D*_v_ (nm)	*A*_eff_ (%)	*N*	*P* (%)
2.5	326 ± 92	350 ± 165	97	548	36
5.0	213 ± 67	199 ± 118	95	250	45
10	174 ± 60	147 ± 73	65	175	46

a Nanoemulsion preparation conditions: applied pressure = 20 000 psi for 10 passes using an LV1 microfluidizer. [N.B. *D*_z_ is the z-average hydrodynamic diameter as determined by DLS; *D*_v_ is the volume-average droplet diameter, as determined by analytical centrifugation; *A*_eff_ is the nanoparticle adsorption efficiency, as determined *by* UV GPC;^43^*N* is the number of nanoparticles per droplet; and *P* is the packing efficiency, calculated using a model reported by Balmer et al.^69^]

**Figure 8:**
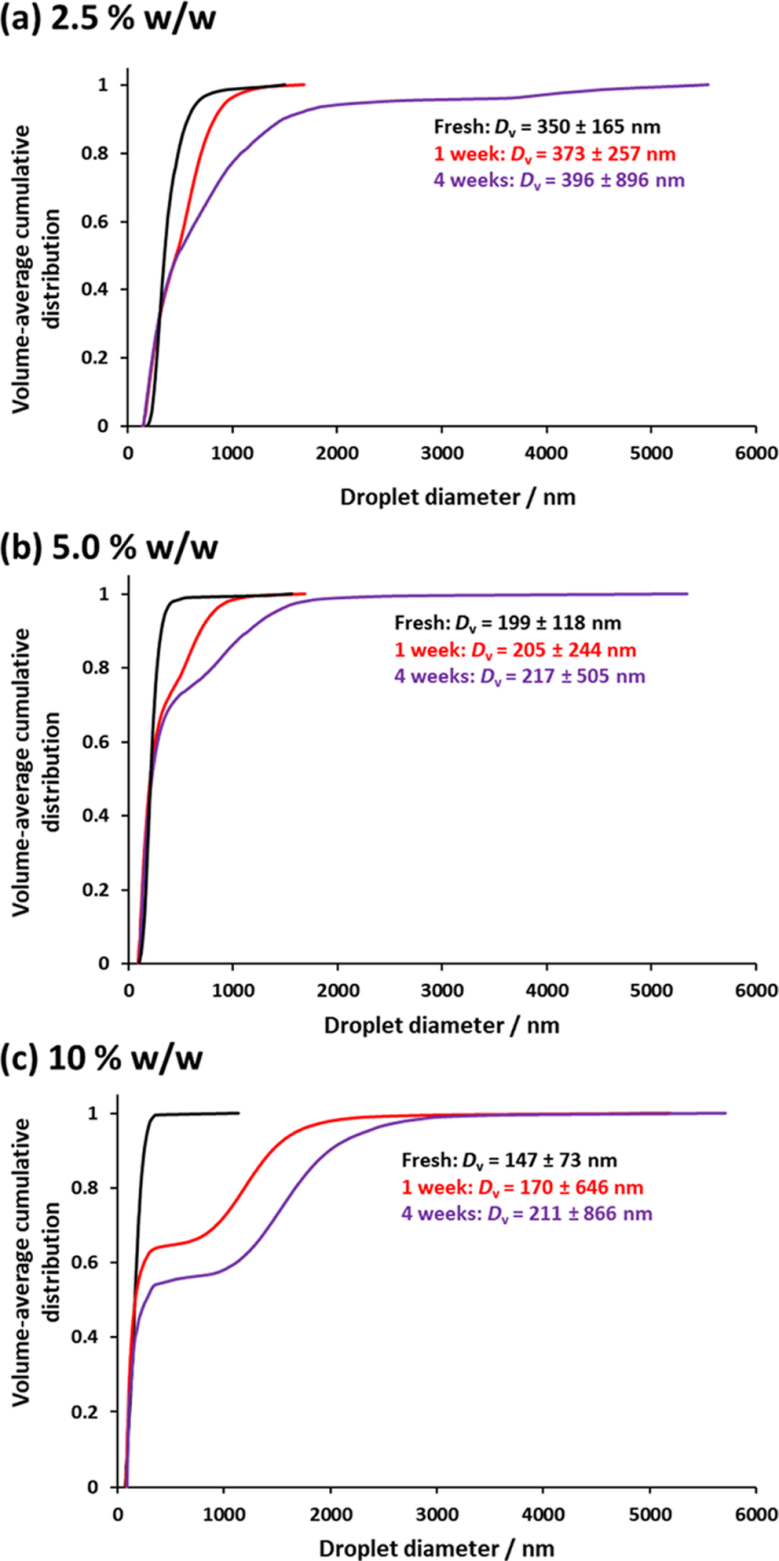
Volume-average cumulative distributions determined by analytical centrifugation for fresh and aged n-dodecane-in-water Pickering nanoemulsions at 20% v/v, prepared using (a) 2.5% w/w, (b) 5.0% w/w, or (c) 10% w/w PGMA_52_–PTFEMA_50_ diblock copolymer nanoparticles. The harmonic mean ± standard deviation is reported in each case. Nanoemulsion preparation conditions: applied pressure = 20 000 psi for 10 passes using an LV1 microfluidizer.

To determine the effect of copolymer concentration on the long-term stability of these Pickering nanoemulsions, their droplet size distributions were monitored by DLS (see Figure S5) and analytical centrifugation (see Figure 8). Perhaps surprisingly, DLS seems to be relatively insensitive to droplet growth over longer time scales compared to analytical centrifugation. Similar observations were reported by Thompson and co-workers^70,71^ but the precise reason for this unexpected discrepancy is still unknown. Although the mean DLS droplet diameter did not increase significantly, the DLS polydispersity (which is a crude measure of the width of the droplet size distribution) increased approximately two-fold after aging each nanoemulsion for four weeks at 20 °C. This is consistent with broadening of the original droplet size distribution.

The analytical centrifugation data confirmed that finer droplets are indeed obtained when using higher nanoparticle concentrations. Moreover, the mean droplet diameter increases on aging each of the three nanoemulsions. This is consistent with Ostwald ripening, which is widely considered to be the dominant mechanism for the destabilization of Pickering nanoemulsions.^71,78^ Moreover, the most significant change in the droplet size distribution was observed for the nanoemulsion prepared using 10% w/w nanoparticles. One likely reason for this is the relatively small droplet radius. According to the Kelvin equation, this leads to higher oil solubility within the aqueous phase^75^
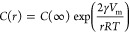
2where *C*(*r*) is the solubility of the spherical droplets dispersed within the continuous phase, *C*(∞) is the bulk phase solubility (i.e., the solubility of an infinitely large droplet), and *V*_m_ is the molar volume of the dispersed phase. This inevitably leads to a faster rate of Ostwald ripening, as indicated by eq 1. The faster rate of Ostwald ripening for nanoemulsions prepared at higher nanoparticle concentrations can also be explained by the presence of excess nanoparticles. Such non-adsorbed nanoparticles accelerate the ripening process by enhancing the rate of mass transport of oil molecules within the aqueous phase. Furthermore, a transition from an initial unimodal size distribution to bimodal droplet size distributions is observed for nanoemulsions prepared using 5.0 and 10% w/w nanoparticles, which agrees with data previously reported by Hunter and Armes.^18^ This suggests that using higher nanoparticle concentrations (more specifically, the presence of excess nanoparticles that typically arise when using higher nanoparticle concentrations) has a detrimental effect on the long-term stability of Pickering nanoemulsions.

The effect of varying the nanoparticle concentration on the adsorption efficiency was also studied over a four-week period. The same three *n*-dodecane-in-water nanoemulsions prepared using 2.5, 5.0, or 10% w/w PGMA_52_–PTFEMA_50_ nanoparticles were used for these experiments. Each nanoemulsion was centrifuged at 13 000 rpm for 15 min to allow creaming of the low-density oil droplets. The underlying aqueous supernatant was then analyzed via UV GPC at a fixed wavelength of 298 nm, and the calibration plot previously constructed for the PGMA_52_–PTFEMA_50_ nanoparticles (Figure S4) was used to determine the concentration of non-adsorbed nanoparticles. The results obtained from these experiments are summarized in Table 2.

**Table 2:** Summary of the Characterization Data Obtained after Aging the Three *n*-Dodecane-in-Water Pickering Nanoemulsions Prepared Using 2.5% w/w PGMA_52_–PTFEMA_50_ Nanoparticles for up to 4 weeks at 20 °C^a^

time (week)	*D*_v_ (nm)	*A*_eff_ (%)	*N*	*P* (%)
fresh	350	97	581	43
1	373	97	718	46
2	375	96	724	46
3	379	95	742	46
4	396	97	876	50

a Nanoemulsion preparation conditions: applied pressure = 20 000 psi for 10 passes using an LV1 microfluidizer. [N.B. *D*_v_ is the volume-average droplet diameter determined by analytical centrifugation; *A*_eff_ is the nanoparticle adsorption efficiency determined using UV GPC;^43^*N* is the number of nanoparticles per droplet; and *P* is the packing efficiency, calculated using a model reported by Balmer et al.^69^]

Almost no change in adsorption efficiency was observed for the *n*-dodecane-in-water nanoemulsion prepared at a nanoparticle concentration of 2.5% w/w. At first sight, this does not appear to be consistent with the significant increase in droplet diameter that is observed over time for this nanoemulsion. Larger droplets should lead to a reduction in the overall surface area, resulting in a reduction in nanoparticle surface coverage and hence a lower adsorption efficiency. However, Ostwald ripening also results in preferential dissolution of the smaller oil droplets according to the Kelvin equation. As these finer droplets shrink over time, nanoparticle desorption from the oil–water interface should occur owing to the reduction in interfacial area. Subsequently, these nanoparticles adsorb onto the growing larger droplets to maintain their original high surface coverage. This explains the constant adsorption efficiency (∼97%) that is observed over time. Since the volume-average droplet diameter increases over time and the adsorption efficiency remains essentially unchanged, the number of nanoparticles per droplet, *N*, necessarily increases. According to Table 2, there is also a modest increase in the packing efficiency, *P*. In principle, the lower interfacial curvature of the growing oil droplets enables more efficient nanoparticle packing at the oil/water interface.

### Effect of Temperature on the Stability of Pickering Nanoemulsions

Several studies have examined the effect of storage temperature on the stability of surfactant-stabilized nanoemulsions.^73,76−79^ For example, Delmas et al.^73^ reported that the rate of Ostwald ripening, ω, of O/W nanoemulsions stabilized using a non-ionic surfactant followed Arrhenius behavior according to eq 3:
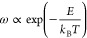
3

**Figure 9:**
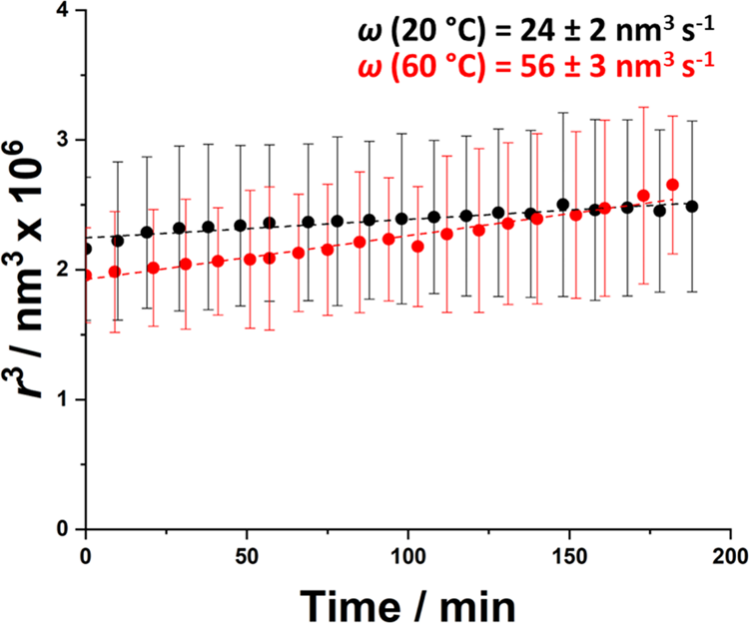
Variation in the cube of the mean droplet radius (*r*^3^) over time as determined by DLS studies of aging *n*-dodecane-in-water Pickering nanoemulsions at either 20 °C (black data points) or 60 °C (red data points). Nanoemulsion preparation conditions: 7.0% w/w PGMA_52_–PTFEMA_50_ nanoparticles; applied pressure = 20 000 psi; 10 passes through an LV1 microfluidizer.

As far as we are aware, there have been no reports of the effect of aging Pickering nanoemulsions at elevated temperature. In view of this gap in the literature, we decided to study the effect of temperature on the rate of Ostwald ripening of *n*-dodecane-in-water Pickering nanoemulsions. The *z*-average diameter of a freshly prepared *n*-dodecane-in-water nanoemulsion diluted to 1% v/v was monitored at 10 min intervals while aging at either 20 or 60 °C using DLS. This technique was preferred to analytical centrifugation for such experiments because it enabled many measurements to be recorded over a relatively short time scale. The cube of the mean droplet radius (*r*^3^) increased approximately linearly over time for the first 160 min (see Figure 9).

This is consistent with LSW theory for Ostwald ripening, see eq 1.^37,70,71^ From the gradients of these two linear plots, the rate of Ostwald ripening ω is calculated to be 24 ± 2 nm^3^ s^–1^ at 20 °C and 56 ± 3 nm^3^ s^–1^ at 60 °C. Moreover, Kabalnov et al. reported that experimental ω values are systematically higher by a factor of ∼2.5 compared to those calculated using LSW theory.^80^ This discrepancy was attributed to the Brownian motion of emulsion droplets because LSW theory is derived for droplets trapped within a solid matrix. Thus the above experimental ω value observed at 20 °C is comparable to the theoretical value (12 nm^3^ s^–1^) calculated for *n-*dodecane droplets at the same temperature.^37^ Furthermore, the more than two-fold increase in ω observed at 60 °C is comparable to data reported by Delmas and co-workers for surfactant-stabilized O/W nanoemulsions aged at various temperatures ranging from 25 to 60 °C.^73^ This observation was attributed to the temperature-sensitive nature of both the solubility and diffusivity of the dispersed phase.

**Figure 10:**
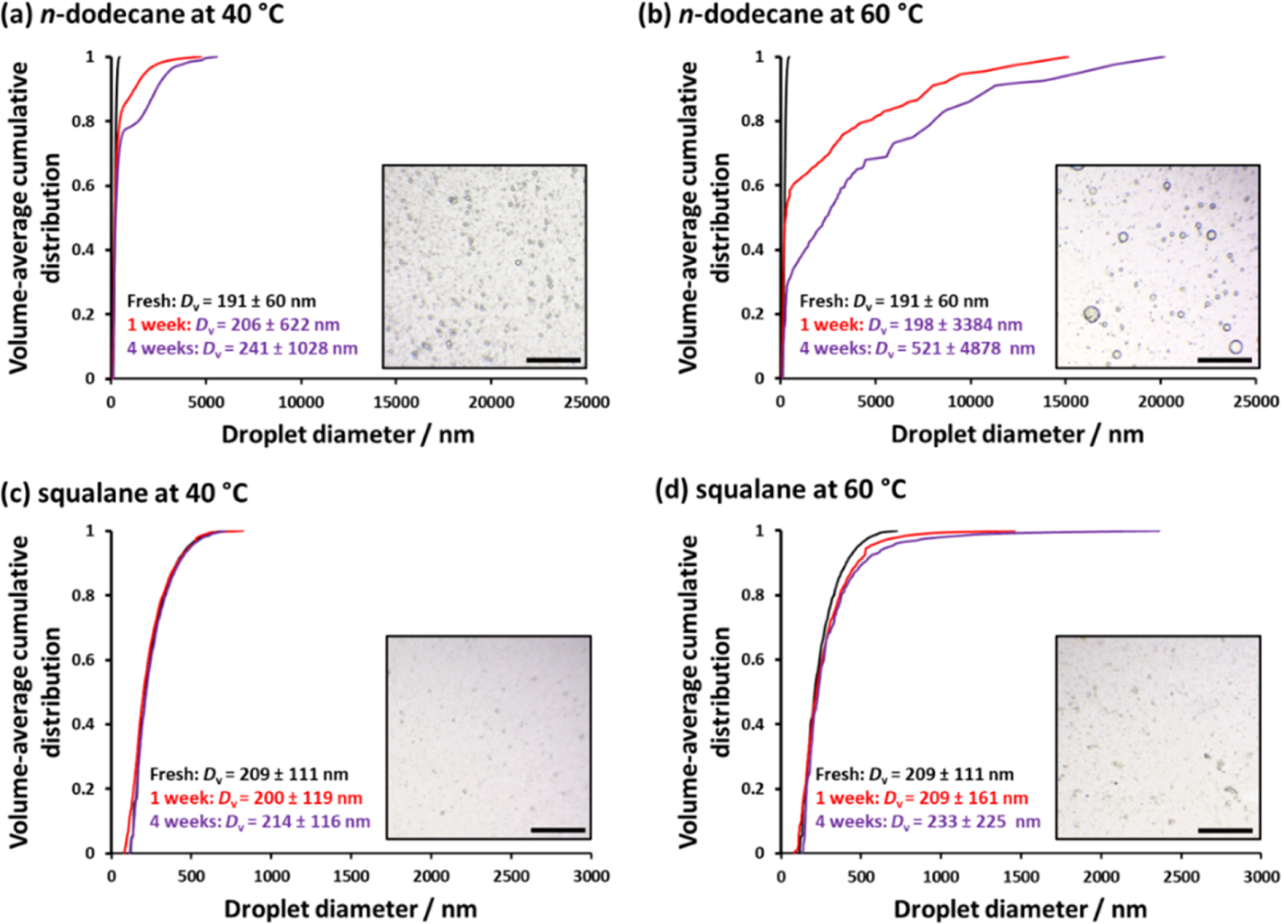
Volume-average cumulative droplet size distributions determined by analytical centrifugation for fresh and aged Pickering nanoemulsions prepared at 20% v/v using either (a, b) *n*-dodecane or (c, d) squalane as the droplet phase after storage at either 40 or 60 °C. The harmonic mean ± standard deviation is reported for the fresh and aged nanoemulsions. Insets show optical microscopy images recorded for each aged nanoemulsion after 4 weeks (scale bars represent 100 μm). Nanoemulsion preparation conditions: applied pressure = 20 000 psi for 10 passes using an LV1 microfluidizer.

To further explore the effect of temperature on Ostwald ripening, we studied the *long-term* stability of the O/W Pickering nanoemulsions prepared using either *n*-dodecane or squalane. Accordingly, freshly prepared nanoemulsions were stored at either 40 or 60 °C and the droplet size distribution of each nanoemulsion was monitored over a four-week period using analytical centrifugation. Figure 10 shows the temperature-dependent stability of the Pickering nanoemulsions. Clearly, aging at 40 °C leads to the evolution of broader *n*-dodecane droplet size distributions over time. Moreover, this instability is much more discernible at 60 °C, as confirmed by optical microscopy studies (see inset images recorded for the final aged nanoemulsions). In contrast, there is essentially no change in the droplet size distributions recorded for squalane-in-water nanoemulsions aged for four weeks at 40 °C, although some increase in droplet size is detected at 60 °C.

Schatzberg^81^ reported that the aqueous solubility of *n*-dodecane at 40 °C is almost twice that at 25 °C and that this temperature dependence is consistent with theoretical aqueous solubilities calculated using the Hildebrand equation.^82^ Moreover, Stevenson and co-workers determined the aqueous solubility of squalane at elevated temperature (above 600 K) and pressure (greater than 150 bar).^83^ Linear extrapolation of these data suggests that the aqueous solubility of squalane at ambient pressure is approximately 0.011 μg dm^–3^ at 20 °C, 0.014 μg dm^–3^ at 40 °C, and 0.016 μg dm^–3^ at 60 °C.

Hence the observed differing nanoemulsion stabilities simply reflect the higher aqueous solubility of *n*-dodecane compared to squalane at any given temperature plus the known temperature-dependent aqueous solubility of each oil. These experimental observations are consistent with an Ostwald ripening mechanism for droplet growth.

## Conclusions

Well-defined PGMA_52_–PTFEMA_50_ spherical nanoparticles were synthesized via aqueous PISA for the preparation of O/W Pickering nanoemulsions by (i) high-shear homogenization and (ii) high-pressure microfluidization. The long-term stability of the resulting *n*-dodecane-in-water and squalane-in-water Pickering nanoemulsions was studied over a four-week period at 20–60 °C using analytical centrifugation. The mean droplet size increased much more rapidly when using *n*-dodecane compared to squalane. This is attributed to the higher aqueous solubility of the former oil, which leads to a faster rate of Ostwald ripening. The addition of 20 to 40% (v/v) squalane to *n*-dodecane droplets prior to emulsification leads to more stable Pickering nanoemulsions. A series of *n*-dodecane-in-water Pickering nanoemulsions were prepared using 4.0, 7.0, or 10% w/w PGMA_52_–PTFEMA_50_ nanoparticles to investigate the effect of nanoparticle concentration on adsorption efficiency. UV GPC was used to determine the fraction of non-adsorbed nanoparticles for each nanoemulsion. Increasing the nanoparticle concentration reduced the mean droplet size and hence lowered the adsorption efficiency from almost 100% (when using 4.0% w/w nanoparticles) to 77% (when using 10% w/w nanoparticles). The number of nanoparticles adsorbed onto each droplet was also reduced at higher nanoparticle concentrations, owing to the formation of smaller droplets. DLS and analytical centrifugation studies both show that the rate of Ostwald ripening for *n*-dodecane-in-water Pickering nanoemulsions is temperature-dependent, which is consistent with LSW theory. More specifically, the rate of Ostwald ripening at 60 °C is more than twice that at 20 °C. In contrast, squalane-in-water nanoemulsions are significantly more resistant to Ostwald ripening when aged at the same temperature over a period of four weeks. This simply reflects the differing temperature-dependent aqueous solubilities of these two model oils.

## Supporting Information Available

^1^H NMR spectra for the PGMA_52_ precursor and ^1^H NMR and ^19^F NMR spectra for the PGMA_52_–PTFEMA_50_ diblock copolymer; DMF GPC curves for the PGMA_52_ precursor and PGMA_52_–PTFEMA_50_ diblock copolymer; UV GPC calibration plot for the PGMA_52_–PTFEMA_50_ diblock copolymer; DLS data recorded for aging *n*-dodecane-in-water Pickering nanoemulsions prepared using various concentrations of PGMA_52_–PTFEMA_50_ nanoparticles; details of the core–shell and spherical micelle model scattering models used for the SAXS analysis (PDF)
