# Supplementary material for: Effect of Temperature, Oil Type, and Copolymer Concentration on the Long-Term Stability of Oil-in-Water Pickering Nanoemulsions Prepared Using Diblock Copolymer Nanoparticles
Source: Langmuir. 2024 Feb 5;40(7):3702–14. doi: 10.1021/acs.langmuir.3c03423 (PMC10883058; doi:10.1021/acs.langmuir.3c03423)
Supplement: Supplementary file 1 — la3c03423_si_001.pdf [file la3c03423_si_001.pdf]

Supporting Information for:

Effect of temperature, oil type and copolymer concentration on the long-term stability of oil-in-water Pickering nanoemulsions prepared using diblock copolymer nanoparticles

*Saul J. Hunter,**,‡ *Priyanka Chohan,*† *Spyridon Varlas*† *and Steven P. Armes**,†

†*Department of Chemistry, Dainton Building, University of Sheffield, Brook Hill, Sheffield, South Yorkshire, S3 7HF, UK.*

‡*School of Chemistry, Joseph Banks Laboratories, University of Lincoln, Brayford Pool, Lincoln, LN6 7TS, UK.*

Table of Contents

Figure S1 Assigned ^{1}H NMR spectra recorded for a $PGMA_{52}$ precursor. S2

Figure S2 Assigned ^{1}H and ^{19}F NMR spectra recorded for $PGMA_{52}$-$PTFEMA_{50}$ diblock copolymer. S3

Figure S3 DMF GPC curves recorded for the $PGMA_{52}$ precursor and the corresponding $PGMA_{52}$–$PTFEMA_{50}$ diblock copolymer. S4

Figure S4 UV GPC calibration plot constructed for varying concentrations of $PGMA_{52}$–$PTFEMA_{50}$ diblock copolymer. S5

Figure S5 DLS particle size distributions recorded for a series of n-dodecane-in-water nanoemulsions prepared using different copolymer concentrations. S6

Structural Models for Small-Angle X-ray Scattering (SAXS) Analysis S7

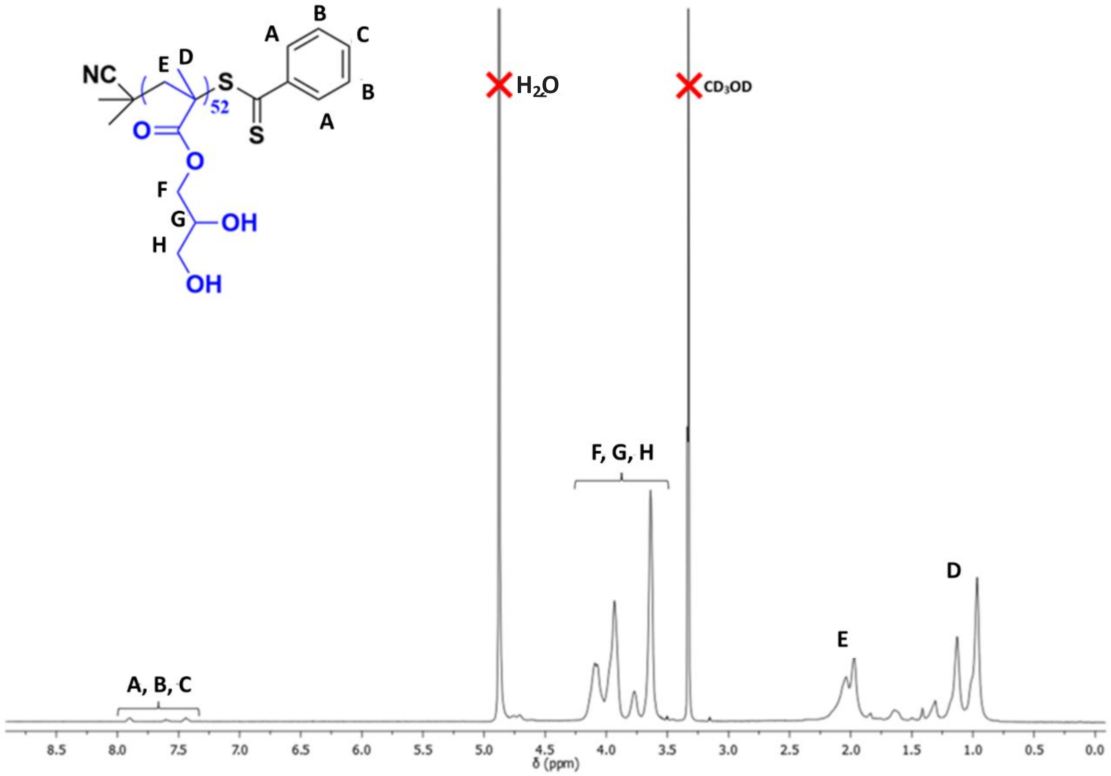

Figure S1. Assigned ^{1}H NMR spectra (CD_3OD) recorded for a $PGMA_{52}$ precursor.

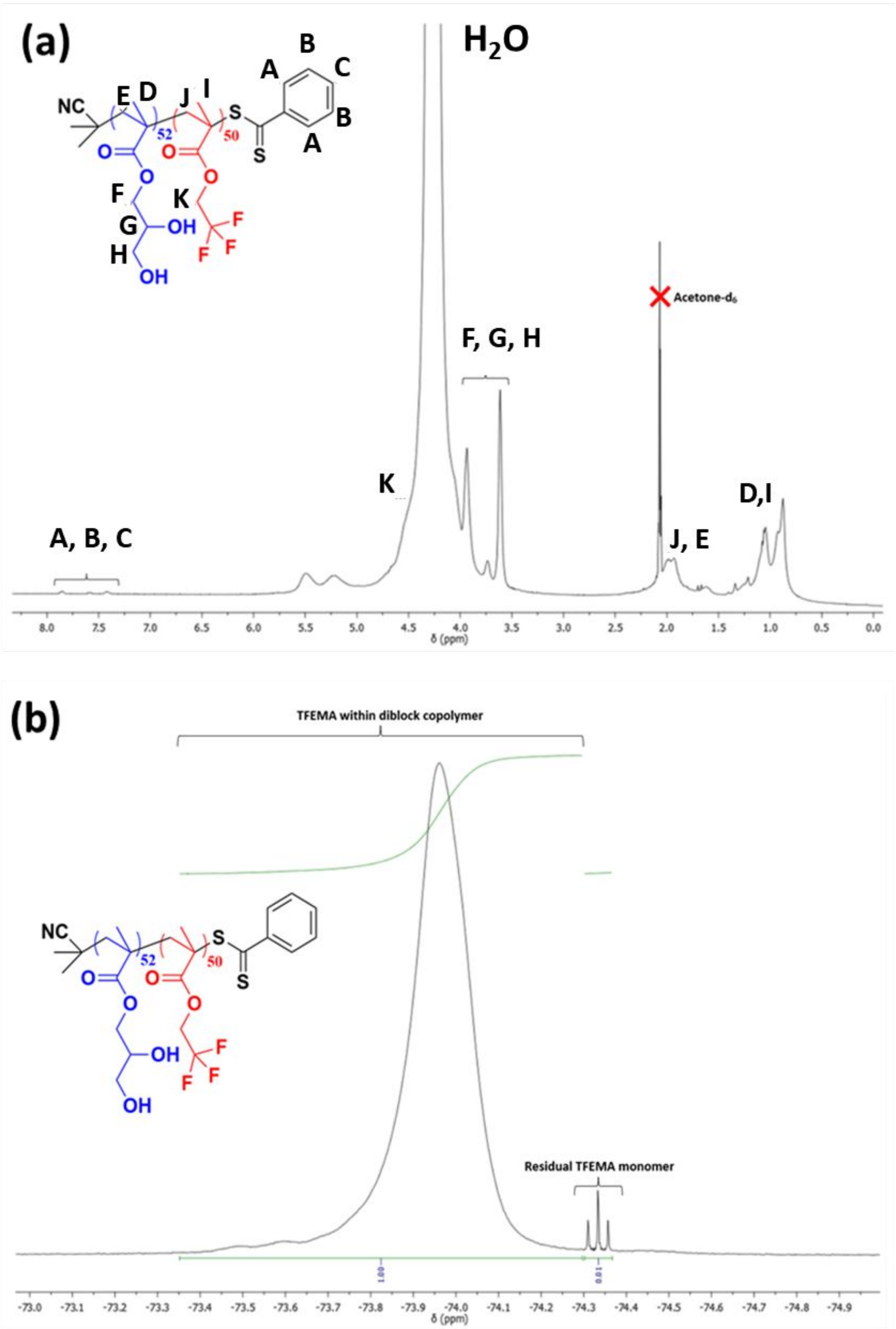

Figure S2. (a) Assigned ^{1}H NMR spectra (CD_3OD) recorded for a $PGMA_{52}$-$PTFEMA_{50}$ diblock copolymer. (b) ^{19}F NMR spectrum (d_6-acetone) recorded for a $PGMA_{52}$–$PTFEMA_{50}$ diblock copolymer. Comparison of the integrated PTFEMA and residual TFEMA monomer signals indicated a TFEMA conversion of more than 99%.

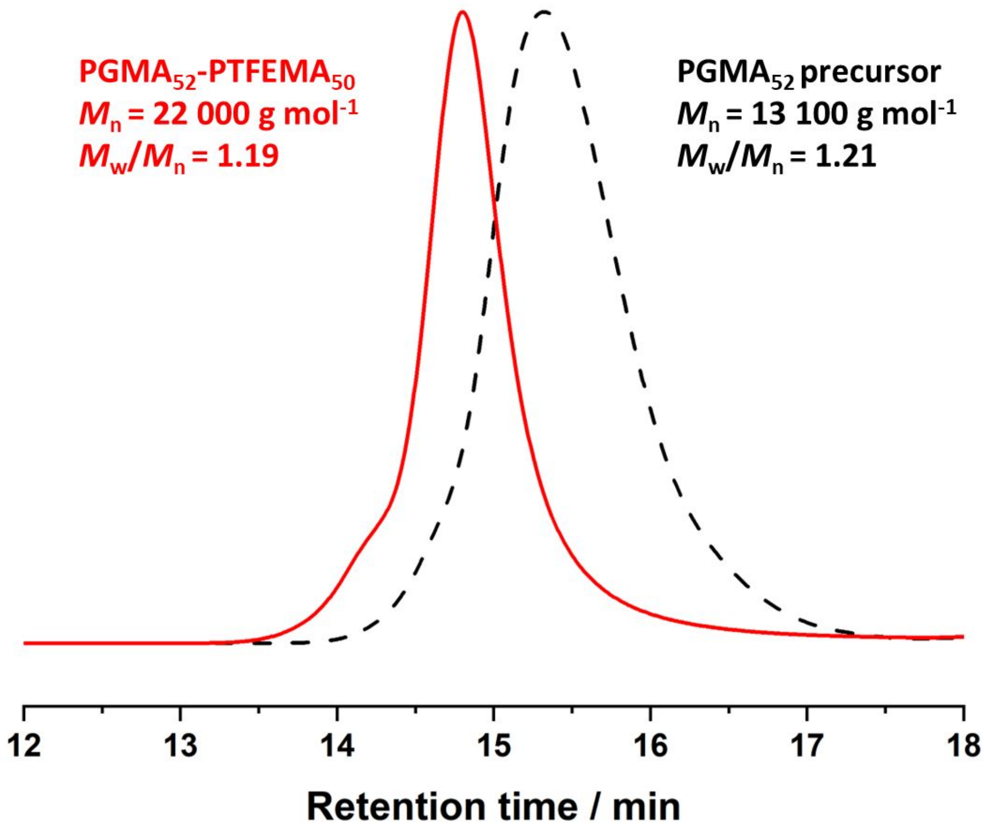

Figure S3. DMF GPC curves (UV detector; λ = 298 nm) recorded for the $PGMA_{52}$ precursor (black trace) and the corresponding $PGMA_{52}$–$PTFEMA_{50}$ diblock copolymer (red trace) prepared via RAFT solution polymerization and RAFT aqueous emulsion polymerization, respectively.

Table S1. Structural parameters obtained by SAXS analysis of 1.0% w/w $PGMA_{52}$-$PTFEMA_{50}$ nanoparticles and 1.0% v/v Pickering nanoemulsions comprising squalane droplets prepared $PGMA_{52}$-$PTFEMA_{50}$ nanoparticles.

	Spherical micelle model				Core-shell model			Hard-sphere structure factor	
	D_s / nm	R_g / nm	x_{sol}	φ_s	D_{core} / nm	T_{shell} / nm	φ_{CS}	D_{HS} / nm	φ_{HS}
PGMA-PTFEMA nanoparticles	21 ± 1	2.2	0.01	0.008	-	-	-	-	-
Pickering nanoemulsion	21 ± 1	2.2	0.01	0.001	184 ± 45	8	0.009	30	0.15

D_s = copolymer nanoparticle diameter; R_g = radius of gyration of $PGMA_{52}$ stabilizer block; x_{sol} = degree of solvation of PTFEMA core; D_{core} = mean core diameter; T_s = mean shell thickness; D_{HS} = hard-sphere radius of packed copolymer nanoparticles; c_{HS} = volume fraction of packed copolymer nanoparticles; ξ = effective scattering length density.

Parameters used for modeling are as follows: $\xi_{solvent} = 9.42 \times 10^{10}$ cm^{-2}; $\xi_c = 7.68 \times 10^{10}$ cm^{-2}; $\xi_{shell} = 10.85 \times 10^{10}$ cm^{-2}. ξ_{shell} was calculated by averaging the scattering length densities of the PGMA stabilizer block (11.94×10^{10} cm^{-2}), the PTFEMA core-forming block (12.76×10^{10} cm^{-2}) and the solvent (water) based on the copolymer composition and packing efficiency of the copolymer nanoparticles at the surface of the oil droplets.

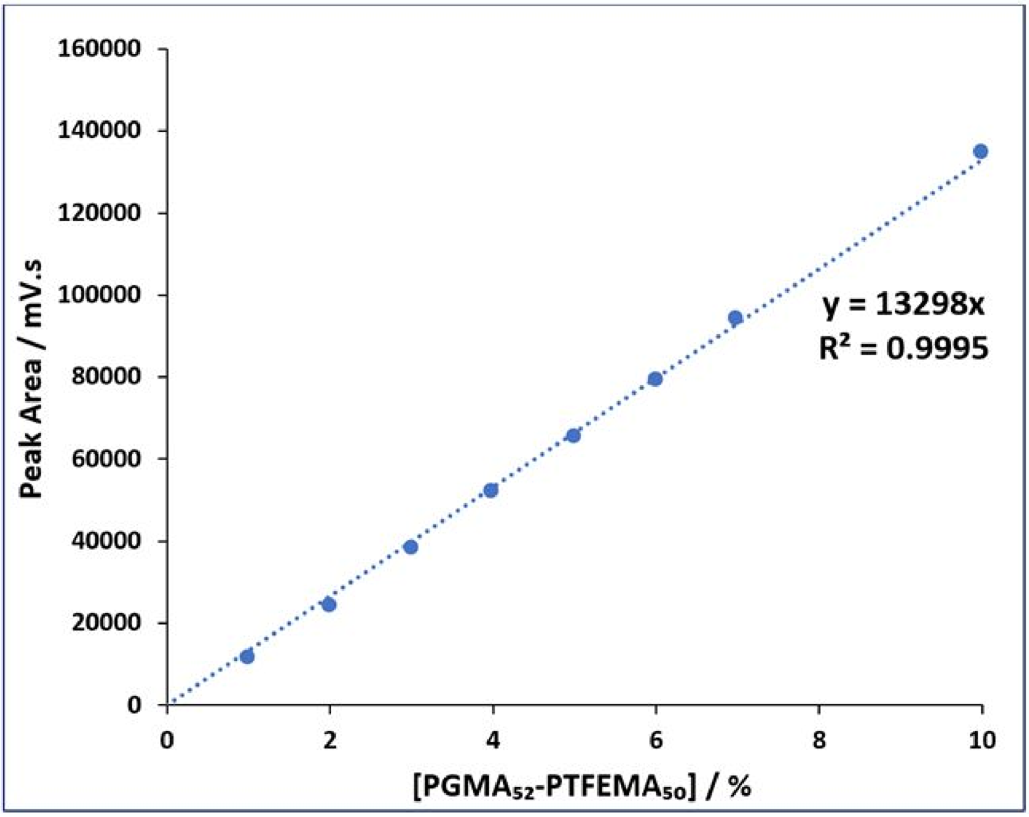

Figure S4. UV GPC calibration plot constructed for varying concentrations of $PGMA_{52}$–$PTFEMA_{50}$ diblock copolymer nanoparticles recorded at a wavelength of 298 nm.

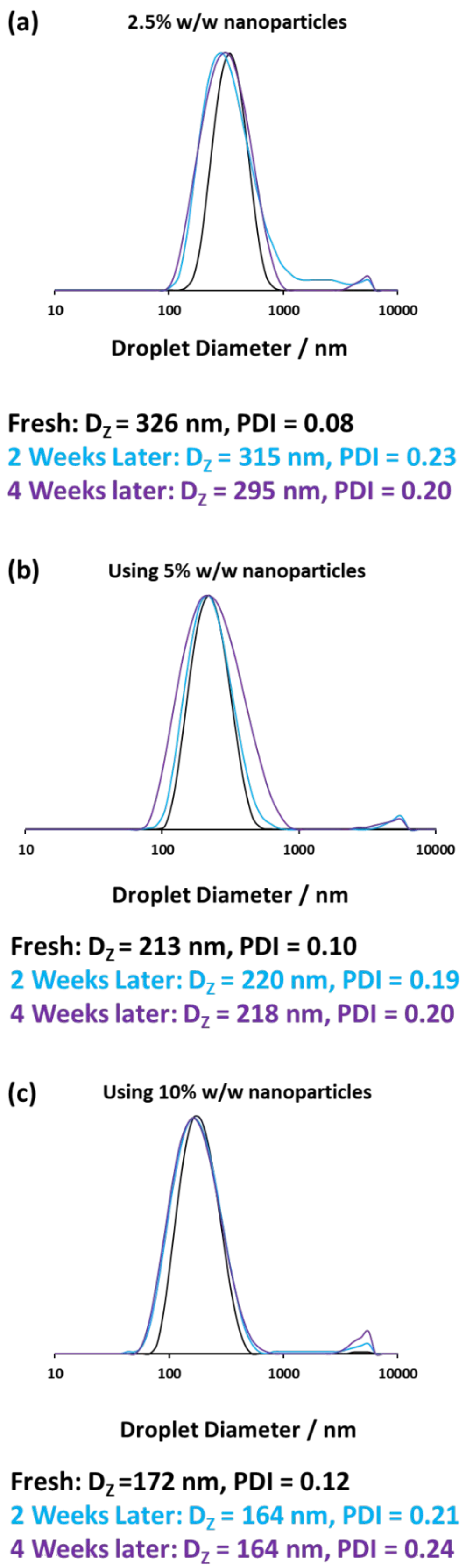

Figure S5. DLS particle size distributions recorded for a series of *n*-dodecane-in-water nanoemulsions prepared using (a) 2.5% w/w (b) 5.0% w/w or (c) 10% w/w $PGMA_{52}$–$PTFEMA_{50}$ diblock copolymer nanoparticles during ageing for four weeks at 20 °C.

Structural Models Used for Small-Angle X-ray Scattering (SAXS) Analysis

Spherical micelle and core-shell scattering models were taken from the literature[1-5] and modified as indicated below. In general, the intensity of X-rays scattered by a dispersion of nanoparticles [usually represented by the scattering cross section per unit sample volume, $\frac{d\Sigma}{d\Omega}(q)$] can be expressed as:

$$\frac{d\Sigma}{d\Omega}(q) = NS(q)\int_0^\infty \ldots \int_0^\infty F(q,r_1,\ldots,r_k)^2\Psi(r_1,\ldots,r_k)dr_1,\ldots,dr_k \qquad \mathbf{1}$$

where $F(q,r_1,\ldots,r_k)$ is the form factor, $r_1,\ldots,r_k$ is a set of k parameters describing the structural morphology, $\Psi(r_1,\ldots,r_k)$ is the distribution function, $S(q)$ is the structure factor and N is the nanoparticle number density per unit volume expressed as:

$$N = \frac{\varphi}{\int_0^\infty \ldots \int_0^\infty V(r_1,\ldots,r_k)\Psi(r_1,\ldots,r_k)dr_1,\ldots,dr_k} \qquad \mathbf{2}$$

where $V(r_1,\ldots,r_k)$ is the volume of the nanoparticle and φ is the volume fraction of nanoparticles.

Given the relatively low nanoparticle concentration, the structure factor term in Equation S1 was assumed to be unity [$S(q) = 1$].

Spherical Micelle Model

The spherical micelle form factor for Equation S1 is given by:[3]

$$F_{s_{mic}}(q,r_1) = N_s^2(r_1)\beta_s^2A_s^2(q,r_1) + N_s(r_1)\beta_c^2F_c(q,R_g) + N_s(r_1)[N_s(r_1) - N_s^2(r_1)\beta_s\beta_cA_s(q,r_1)A_c(q) \qquad \mathbf{3}$$

where r_1 is the radius of the sphere core and R_g is the radius of gyration of the coronal steric stabilizer block (in this case, $PGMA_{52}$). The X-ray scattering length contrasts for the core and corona blocks are given by $\beta_s = V_s(\xi_s - \xi_{sol})$ and $\beta_c = V_c(\xi_c - \xi_{sol})$ respectively. Here, ξ_s,

ξ_c and ξ_{sol} are the X-ray scattering length densities of the core block (ξ_{PTFEMA}= 12.76 x 10^{10} cm^{-2}), corona block (ξ_{PGMA}= 11.94 x 10^{10} cm^{-2}) and solvent (water) (ξ_{sol}= 9.42 x 10^{10} cm^{-2}), respectively. V_s and V_c are the volumes of the PTFEMA core block and the PGMA corona block, respectively. These volumes were calculated using $V = \frac{M_{n,\,pol}}{N_A \rho}$ where the mass density of a PTFEMA homopolymer was previously reported (ρ_{PTFEMA} = 1.47 g cm^{-3})[6] and the density of PGMA was taken to be 1.31 g cm^{-3}.[4] $M_{n,\,pol}$ corresponds to the number-average molecular weight of the diblock copolymer chains determined by ^{1}H NMR spectroscopy. The sphere form factor amplitude is used for the amplitude of the core self-term:

$$A_s(q,r_1) = \Phi(qr_1)\exp\left(-\frac{q^2\sigma^2}{2}\right) \qquad \mathbf{4}$$

where $\Phi(qr_1) = \frac{3[\sin(qr_1) - qr_1\cos(qr_1)]}{(qr_1)^3}$. A sigmoidal interface between the two blocks was assumed for the spherical micelle form factor (Equation S4). This is described by the exponent term with a width σ accounting for a decaying scattering length density at the micellar interface. This σ value was fixed at 0.25 nm during fitting.

The form factor amplitude of the spherical micelle corona is:

$$A_c(q) = \frac{\int_{r_1}^{r_1+2s} \mu_c(r)\frac{\sin(qr)}{qr} r^2 dr}{\int_{r_1}^{r_1+2s} \mu_c(r) r^2 dr} exp\left(-\frac{q^2\sigma^2}{2}\right) \qquad \mathbf{5}$$

The radial profile, $\mu_c(r)$, can be expressed by a linear combination of two cubic b splines, with two fitting parameters *s* and *a* corresponding to the width of the profile and the weight coefficient, respectively. The self-correlation term for the corona block is given by the Debye function:

$$F_{\mathrm{c}}(q,R_{\mathrm{g}}) = \frac{2\left[\exp\left(-q^2R_g^2\right) - 1 + q^2R_g^2\right]}{q^4R_g^4} \quad \textbf{6}$$

The structure factor in Equation S2, $S_1(q)$, is usually expressed for interacting spherical micelles as:

$$S_1(q) = 1 + \frac{A_1^{av}(q,r_{11})^2\left[\left(S_{\mathrm{PY}}(q, R_{\mathrm{PY}}, f_{PY}\right) - 1\right]}{F_1(q,r_{11})} \quad \textbf{7}$$

Herein the form factor of the average radial scattering length density distribution of micelles is expressed as $A_{\mathrm{av}}(q, r_1) = N_{\mathrm{s}}[\beta_{\mathrm{s}}A_{\mathrm{s}}(q, r_{11}) + \beta_{\mathrm{c}}A_{\mathrm{c}}(q)]$ and $S_{\mathrm{PY}}(q, R_{\mathrm{PY}}, f_{\mathrm{PY}})$ is a hard-sphere interaction structure factor solved using the Percus-Yevick closure relation,[7] where R_{PY} is the interaction radius and f_{PY} is the hard-sphere volume fraction. For dilute dispersions of micelles it is assumed that $S_{\mathrm{PY}}(q, R_{\mathrm{PY}}, f_{\mathrm{PY}}) = 1$.

A polydispersity for one parameter (r_1) is assumed for the micelle model, which is described by a Gaussian distribution. Thus, the polydispersity function in Equation S1 can be represented as:

$$\Psi(r_1) = \frac{1}{\sqrt{2\pi\sigma_{R_{\mathrm{s}}}{}^2}} exp\left(-\frac{(r_1 - R_{\mathrm{s}})^2}{2\sigma_{R_{\mathrm{s}}}{}^2}\right) \quad \textbf{7}$$

where R_{s} is the mean spherical micelle core radius and $\sigma_{R_{\mathrm{s}}}$ is its standard deviation. In accordance with Equation S2, the number density per unit volume for the micelle model is expressed as:

$$N = \frac{\varphi}{\int_0^\infty V(r_1)\Psi(r_1)dr_1} \quad \textbf{8}$$

where φ is the total volume fraction of copolymer in the spherical micelles and $V(r_1)$ is the total volume of copolymer in a spherical micelle $V(r_1) = (V_s + V_c)N_s(r_1)$.

Assuming that the projected contour length of a PGMA monomer is 0.255 nm (two C-C bonds in all- trans conformation), the total contour length of a $PGMA_{52}$ block, L_{PGMA52} = 52 x 0.255 nm = 13.26 nm. Given a mean Kuhn length of 1.53 nm (based on the known literature value for PMMA) an estimated unperturbed radius of gyration, R_g = (13.26 x 1.53/6)0.5 = 1.83 nm is calculated. The data fit to the SAXS pattern recorded for $PGMA_{52}$-$PTFEMA_{50}$ spheres using the spherical micelle suggested that the experimental R_g for the corona PGMA block (2.20 nm) is physically reasonable, since it is close to this theoretical estimate.

Core-Shell Model

Following our prior study of the characterisation of core-shell nanocomposite particles comprising polymer latex cores and particulate silica shells,[5] the SAXS data recorded for o/w Pickering nanoemulsions were analysed using a two-population model (n = 2). Population 1 (i = 1) is represented by core-shell spheres, where the cores comprise the oil or water droplets and the adsorbed layer of nanoparticles form the shell. The particulate nature of the shell is described by spherical micelles (see above), which corresponds to population 2 (i = *2*).

Core-Shell Particle Model

The following functions and parameters were used for the core-shell particle (i = 1) model:

$$F_1(q,r_{11}) = V_{\text{total}}(\xi_{\text{shell}} - \xi_{\text{sol}})\Phi[q(r_{11} + T_{\text{shell}})] + V_{\text{core}}(\xi_{\text{core}} - \xi_{\text{shell}})\Phi(qr_{11}) \quad \mathbf{9}$$

where

$$\Phi(x) = \frac{3[\sin(x) - x\cos(x)]}{(x)^3} \quad \mathbf{10}$$

and r_{11} is the core radius and T_{shell} is the shell thickness. $V_{\text{total}} = \frac{4}{3}\pi(r_{11} + T_{\text{shell}})^3$ and $V_{\text{core}} = \frac{4}{3}\pi r_{11}^3$ are volumetric parameters for the core-shell particles, while ξ_{core}, ξ_{shell} and ξ_{sol} are the scattering length densities for the droplet core (ξ_{squalane} = 7.63 x 10^{10} cm^{-2}), ξ_{shell}= 10.85 × 10^{10} cm^{-2}), for the particulate shell and for the surrounding medium (ξ_{H2O} = 9.42 x 10^{10} cm^{-2}), respectively. V_{shell} and V_{core} denote the volume of the nanoparticle shell and the volume of the squalane core for the nanoparticle-stabilized oil droplets, respectively.

Dispersity of only one parameter (the particle core radius) was considered in the SAXS analysis. It was expressed by a Gaussian distribution:

$$\Psi_1(r_{11}) = \frac{1}{(2\pi\sigma_{R_c}{}^2)^{\frac{1}{2}}}\exp\left[\frac{-(r_{11} - R_s)^2}{2\sigma_{R_c}{}^2}\right] \quad \textbf{11}$$

where R_s is the mean core radius and σ_{Rc} is the standard deviation of the droplet core radius. The number density for the first population is expressed as:

$$N_1 = \frac{\varphi_{\text{droplet}}}{\int_0^\infty V(r_{11})\, \Psi_1(r_{11}) \mathrm{d}r_{11}} \quad \textbf{12}$$

where φ_{droplet} is the relative volume fraction of the core-shell nanoemulsion droplets. In all cases, a dilute dispersion (1% v/v) of nanoemulsions has been used, so the structure factor is set to unity [$S_1(q)$=1].

REFERENCES

1. Bang, J.; Jain, S.; Li, Z.; Lodge, T. P.; Pedersen, J. S.; Kesselman, E.; Talmon, Y., Sphere, Cylinder, and Vesicle Nanoaggregates in Poly(styrene-b-isoprene) Diblock Copolymer Solutions. *Macromolecules* **2006,** *39*, 1199-1208.
2. Pedersen, J. S.; Svaneborg, C., Scattering from block copolymer micelles. *Current Opinion in Colloid & Interface Science* **2002,** *7*, 158-166.
3. Pedersen, J., Form factors of block copolymer micelles with spherical, ellipsoidal and cylindrical cores. *Journal of Applied Crystallography* **2000,** *33*, 637-640.

4. Mable, C. J.; Warren, N. J.; Thompson, K. L.; Mykhaylyk, O. O.; Armes, S. P., Framboidal ABC triblock copolymer vesicles: a new class of efficient Pickering emulsifier. *Chemical Science* **2015,** *6*, 6179-6188.
5. Balmer, J. A.; Mykhaylyk, O. O.; Schmid, A.; Armes, S. P.; Fairclough, J. P. A.; Ryan, A. J., Characterization of Polymer-Silica Nanocomposite Particles with Core–Shell Morphologies using Monte Carlo Simulations and Small Angle X-ray Scattering. *Langmuir* **2011,** *27*, 8075-8089.
6. Akpinar, B.; Fielding, L. A.; Cunningham, V. J.; Ning, Y.; Mykhaylyk, O. O.; Fowler, P. W.; Armes, S. P., Determining the Effective Density and Stabilizer Layer Thickness of Sterically Stabilized Nanoparticles. *Macromolecules* **2016,** *49*, 5160-5171.
7. Percus, J. K., Approximation Methods in Classical Statistical Mechanics. *Phys. Rev. Lett.* **1962,** *8*, 462-463.
